# Oxaliplatin-induced type II hypersensitivity in colorectal cancer: a cohort study on clinical presentation, diagnosis, and management

**DOI:** 10.3389/fphar.2025.1605690

**Published:** 2025-08-29

**Authors:** Paula Vázquez-Revuelta, Ricardo Madrigal-Burgaleta, J. Carlos Ruffinelli, Enric Casanovas, Ana Coloma, Ramon Lleonart

**Affiliations:** ^1^ Drug Hypersensitivity and Desensitization Centre (DHDC), Institut Català d’Oncologia (ICO), Barcelona, Spain; ^2^ Allergy Department, Hospital Universitari de Bellvitge (HUB), Barcelona, Spain; ^3^ Institut d’Investigació Biomèdica de Bellvitge (IDIBELL), Barcelona, Spain; ^4^ Universitat de Barcelona (UB), Barcelona, Spain; ^5^ Allergy and Severe Asthma Service, St Bartholomew’s Hospital, Barts Health NHS Trust, London, United Kingdom; ^6^ Medical Oncology Department, Institut Català d’Oncologia (ICO), Barcelona, Spain; ^7^ Immunohaemathology Laboratory, Banc de Sang I Teixits, Barcelona, Spain; ^8^ Nephrology Department, Hospital Universitari de Bellvitge (HUB), Barcelona, Spain

**Keywords:** allergy, cytopenia, chemotherapy, oncology, oxaliplatin, drug hypersensitivity, immune thrombocytopaenia

## Abstract

**Background:**

Oxaliplatin (OXL) is a key treatment for colorectal cancer but can potentially induce type II hypersensitivity reactions (II-HSRs), leading to immune-mediated cytopenias. The prevalence and management of OXL-induced II-HSRs remain poorly understood, with evidence being mainly anecdotal and lacking a systematic approach. This study examines the prevalence, clinical presentation, diagnosis, and management of OXL-induced II-HSRs in our population.

**Methods:**

We prospectively analysed a cohort of OXL-reactive patients at our Drug Hypersensitivity and Desensitisation Centre between January 2019 and April 2024. Patients with clinical and laboratory findings suggestive of II-HSR were included and classified into acute immune thrombocytopenia (AIT), immune haemolytic anaemia (IHA), Evans syndrome (ES), or drug-induced thrombotic microangiopathy (DITMA). Drug-dependent antibodies (DDAbs) were detected via flow cytometry. Carefully selected patients underwent re-exposure to OXL under allergy care and special safety measures.

**Results:**

Sixteen patients were diagnosed with II-HSRs, with a prevalence of 9.5% among OXL-reactive patients. The mean number of OXL cycles at onset was 20. Atypical hypersensitivity symptoms such as chills, fever, and back pain aided clinical identification. AIT was the most common presentation (56%), followed by ES (38%), and one case of DITMA (6%). DDAbs were detected in 86% of cases, with two patients showing DDAbs to other drugs. Five selected patients were re-exposed to OXL without significant complications.

**Conclusion:**

OXL-induced II-HSRs are rare but pose diagnostic and management challenges. This study shows the importance of early identification, the potential role of DDAbs testing, and the feasibility of re-exposure under controlled conditions in selected patients.

## 1 Introduction

Colorectal cancer (CRC) is a major global public health problem, being the third worldwide leading cancer in men and the second in women, and its incidence is increasing (Ferlay et al., 2015). Oxaliplatin (OXL), a third-generation platinum compound, is a pivotal treatment of adjuvant and metastatic CRC, reducing recurrence risk in stage III after complete resection and improving survival in stage IV (Meyerhardt and Mayer, 2005; Goldberg et al., 2023). Beyond CRC, it is also used off-label in various gastrointestinal and gynaecological cancers (Nehls et al., 2008; Cunningham et al., 2008; Dieras et al., 2002). Once in the bloodstream, OXL is converted into highly reactive metabolites that covalently bind to tumour DNA and blood proteins, inhibiting genetic replication and transcription, which leads to cell destruction (Raymond et al., 1998). The most common and limiting adverse effects of OXL include sensory peripheral neuropathy, gastrointestinal toxicity (nausea-vomiting, diarrhoea or mucositis), and haematological toxicity with cytopenias (Bencardino et al., 2025). Hypersensitivity reactions (HSRs) have been reported in 15% (range 1%–25%) of OXL treatments (Pagani et al., 2022; Doña et al., 2024; Saif, 2006).

Unfortunately, Summaries of Product Characteristics (SPCs) typically do not differentiate between HSR subtypes. Publications from various allergy departments show that most HSRs to OXL involve a type I hypersensitivity mechanism, according to Coombs and Gell classification (Saif, 2006; Jimenez-Rodriguez et al., 2024; Coombs and Gell, 1968), activating mast cells in previously sensitized patients (Pagani et al., 2022; Madrigal-Burgaleta et al., 2022). These type I HSRs (I-HSRs) are immediate reactions that occur during or shortly after infusion and may include urticaria, angioedema, bronchospasm, or even anaphylactic shock (Pagani et al., 2022; Madrigal-Burgaleta et al., 2022; Jutel et al., 2023). Extensive knowledge of the pathophysiology of I-HSRs has led to numerous diagnostic and therapeutic guidelines, with rapid drug desensitisation (RDD) considered as the paramount strategy for patients with I-HSRs to continue their treatment safely (Pagani et al., 2022; Jutel et al., 2023).

OXL can also induce Gell and Coombs type II HSRs (II-HSRs), which involve immune-mediated cytopenias (Coombs and Gell, 1968). II-HSRs are mediated by IgG or IgM antibodies against cellular or extracellular matrix antigens (Jutel et al., 2023; Knol and Gilles, 2022; Bajwa and Mohammed, 2024; Demoly et al., 2014). Their mechanism may involve the complement system (cytotoxic antibodies) or effector lymphocytes (natural killer cells, eosinophils, macrophages, or neutrophils) that bind to antibodies associated with target cells via their Fc gamma fragment (FcγR), inducing cytolysis through an alternative complement pathway (antibody-dependent cellular cytotoxicity) (Jutel et al., 2023). In normal conditions, these antibodies have low affinity for self-cells. However, in the presence of the sensitising drug, their binding affinity increases, resulting in immune recognition and destruction of the targeted cells. This mechanism leads to cell destruction and cytopenia —also known as *allergic cytopenia*—which may involve tissue and organ damage and varying complications (Bencardino et al., 2025; Jutel et al., 2023; Curtis SA. et al., 2018).

II-HSRs are traditionally classified as non-immediate reactions because their most apparent manifestations, such as bleeding or cytopenia, typically appear within 5–7 days post-exposure (Madrigal-Burgaleta et al., 2022; Yang and Castells, 2025). However, studies on OXL-induced II-HSRs indicate that typical symptoms can precede bleeding and may occur during drug infusion. The most common symptoms include light-headedness, lower back pain, chills, fever and nausea-vomiting (Bencardino et al., 2025; Aster and Bougie, 2007; Cobo et al., 2007). Some studies suggest that haemolysis may contribute to lower back pain (Desrame et al., 1999).

Drug-induced myelosuppression or tumour invasion of the bone marrow are the most common causes of cytopenias during chemotherapy, leading to the frequent oversight of II-HSRs in the differential diagnosis (Aster and Bougie, 2007; Curtis et al., 2006). Platelets are the primary targets of drug-induced antibodies; however, drugs can also cause immune haemolytic anaemia and neutropenia through similar mechanisms (Aster and Bougie, 2007). For reasons not fully understood, these patients may present with disseminated intravascular coagulation, renal failure, or thrombotic microangiopathy (Aster and Bougie, 2007). II-HSRs to OXL encompass various clinical presentations depending on the affected cell lines and associated tissue damage, with the most common being acute immune thrombocytopenia (AIT), immune haemolytic anaemia (IHA), Evans syndrome (ES) and drug-induced thrombotic microangiopathy (DITMA) (Bencardino et al., 2025; Curtis SA. et al., 2018; Cobo et al., 2007; Stack et al., 2021; Rousseau et al., 2020; Chen et al., 2004; Malkhasyan et al., 2015; Lucchesi et al., 2013; Niu and Mims, 2012; Suzuki et al., 2013; Forcello et al., 2015; Ito et al., 2012; Mason and Rees, 2011; Saif and Ledbetter, 2009; James et al., 2009; Dahabreh et al., 2006; Erdem et al., 2016). Fatal cases of II-HSRs to OXL have been reported (Desrame et al., 1999; Teng et al., 2011; Baret et al., 2013; Shao and Hong, 2008). These conditions can be clinically indistinguishable, with *in vitro* tests often serving as the only means for differential diagnosis.

A significant challenge in identifying drug-induced II-HSRs is confirming the diagnosis, which requires detecting the presence of drug-dependent reactive autoantibodies (DDAbs) using tools that are not routinely available (Arnold et al., 2013; Arnold et al., 2015). The current diagnosis of II-HSRs to OXL relies on clinical criteria and compatible laboratory parameters (e.g., cytopenias, signs of haemolysis or organ damage indicators) in the absence of more probable diagnoses (Aster and Bougie, 2007; Jardim et al., 2012). The limited available experience hinders our understanding of these reactions, and many pathophysiological mechanisms remain unclear. There are no established guidelines on how to diagnose and manage II-HSRs to OXL (Pagani et al., 2022; Madrigal-Burgaleta et al., 2022; Jutel et al., 2023).

This study aims to characterise the clinical features, diagnostic approach, and therapeutic outcomes of OXL induced II-HSRs over a 5-year period in a specialised allergy-led Drug Hypersensitivity and Desensitisation Centre (DHDC) within a regional reference cancer hospital.

## 2 Methods

### 2.1 Study design

This prospective cohort included all patients referred to the DHDC at the Catalan Institute of Oncology in Barcelona, Spain, from January 2019 to April 2024. We analysed our cohort’s database for patients whose clinical presentation was compatible with II-HSRs to OXL. We selected those patients who experienced an adverse reaction in the context of OXL administration, with clinical presentation or laboratory findings suggestive of a II-HSR. The study adhered to the principles of the Declaration of Helsinki and was approved by the hospital’s Ethics Committee (EOM006/25).

### 2.2 Identification of II-HSRs

Following our standard operating procedures (SOPs), all patients who experienced symptoms compatible with a II-HSR during OXL infusion were closely monitored for a week from reaction onset, with serial blood tests arranged. Diagnosis of a II-HSR was based on clinical and analytical criteria established in previous studies (Bencardino et al., 2025; Aster and Bougie, 2007; Jardim et al., 2012). Once identified, II-HSRs were classified into four subtypes: AIT, IHA, ES, and DITMA, as detailed in Table 1. Section A in the Supplementary Material provides the specific criteria for this classification. Allergy work-up included evaluation of other types of hypersensitivity as part of the differential diagnosis—skin testing with OXL (skin prick testing was conducted using a concentration of 5 mg/mL; if negative, intradermal testing was carried out sequentially at 0.5 mg/mL and 1 mg/mL) and any concomitant drugs implicated in the index reaction, analysis of serum biomarkers of hypersensitivity (e.g., tryptase curve or IL-6), risk stratification, and endophenotyping in accordance with our group’s diagnostic pathways (Vázquez-Revuelta et al., 2020; Martí-Garrido et al., 2020; Vázquez-Revuelta et al., 2022; Revuelta et al., 2023). Skin testing followed European Academy of Allergy and Clinical Immunology (EAACI) standards (Pagani et al., 2022).

**TABLE 1:** Criteria for the identification of II-HSRs.

A. Common symptoms	
Lower back pain Fever Chills Rigors	Nausea-vomiting Dark urine Bleeding Oliguria/ anuria

B. Laboratory Tests
FBC Haemoglobin Reticulocytes (no. and %) Haptoglobin LDH Liver function (bilirubin, ALT, AST, GGT, AP) Renal function (creatinine, GFR) PBS DAT Urine sediment

C. Expected results	
AIT	Acute drop of platelet count (% from baseline^a^) PBS with large platelets and normal morphology
IHA	Anaemia, reticulocytosis, high indirect Bb, high LDH, low haptoglobin, spherocytosis on PBS, positive DAT, haemoglobinuria
ES	Criteria for IHA + AIT (can also cause acute immune neutropenia)
DITMA	Acute drop of platelet count (% from baseline^a^), anaemia, reticulocytosis, high LDH, low haptoglobin, schistocytes on PBS, negative DAT, normal coagulation, increased serum creatinine, proteinuria, bland urine sediment

FBC: full blood count, LDH: lactate dehydrogenase, ALT: alanine aminotransferase, AST: aspartate aminotransferase, GGT: gamma-glutamyl transferase, AP: alkaline phosphatase, GFR: glomerular filtration rate, PBS: peripheral blood smear, DAT: direct antiglobulin test, AIT: acute immune thrombocytopenia, IHA: immune haemolytic anaemia, ES: Evans syndrome, DITMA: Drug-induced thrombotic microangiopathy.

^a^ There is no universally accepted threshold defining a specific percentage drop in platelet count or haemoglobin for the diagnosis of AIT, or DITMA. In this study, a sudden decrease exceeding 30% from baseline—when not attributable to other causes such as sepsis, tumour-related consumption, or bone marrow suppression—was considered clinically meaningful and used to support the diagnosis (see Supplementary Material).

### 2.3 Detection of DDAbs by flow cytometry

Flow cytometry for the detection of platelets-associated DDAbs was performed at the Immunohematology Laboratory of the Banc de Sang i Teixits (Barcelona, Spain). The presence of DDAbs was used as an indicator of type-II hypersensitivity. However, given the low sensitivity of the test with some specific drugs, a negative test did not exclude the diagnosis.

For each patient studied, DDAbs were tested not only against oxaliplatin (OXL) but also against concomitant drugs potentially involved in the reaction, such as leucovorin, bevacizumab or premedication). To optimise test sensitivity, samples were collected within 3 weeks after the acute event, even if thrombocytopenia had already resolved. Longer delays were avoided due to the risk of false-negative results associated with the disappearance of circulating antibodies over time.

Before proceeding to the DDAb detection step, an initial indirect flow cytometry test was systematically performed in the absence of the drug to ensure a negative baseline. This step was essential to guarantee that any reactivity observed was truly drug-dependent. If this preliminary test was positive, the DDAb assay was considered invalid due to the risk of false positives (Arnold et al., 2015). In such cases, testing was postponed, and a new serum sample was obtained at least 6 weeks after discontinuation of the suspected drug. The indirect test was then repeated, and, if the result was negative, DDAb detection could proceed.

**FIGURE 1:**
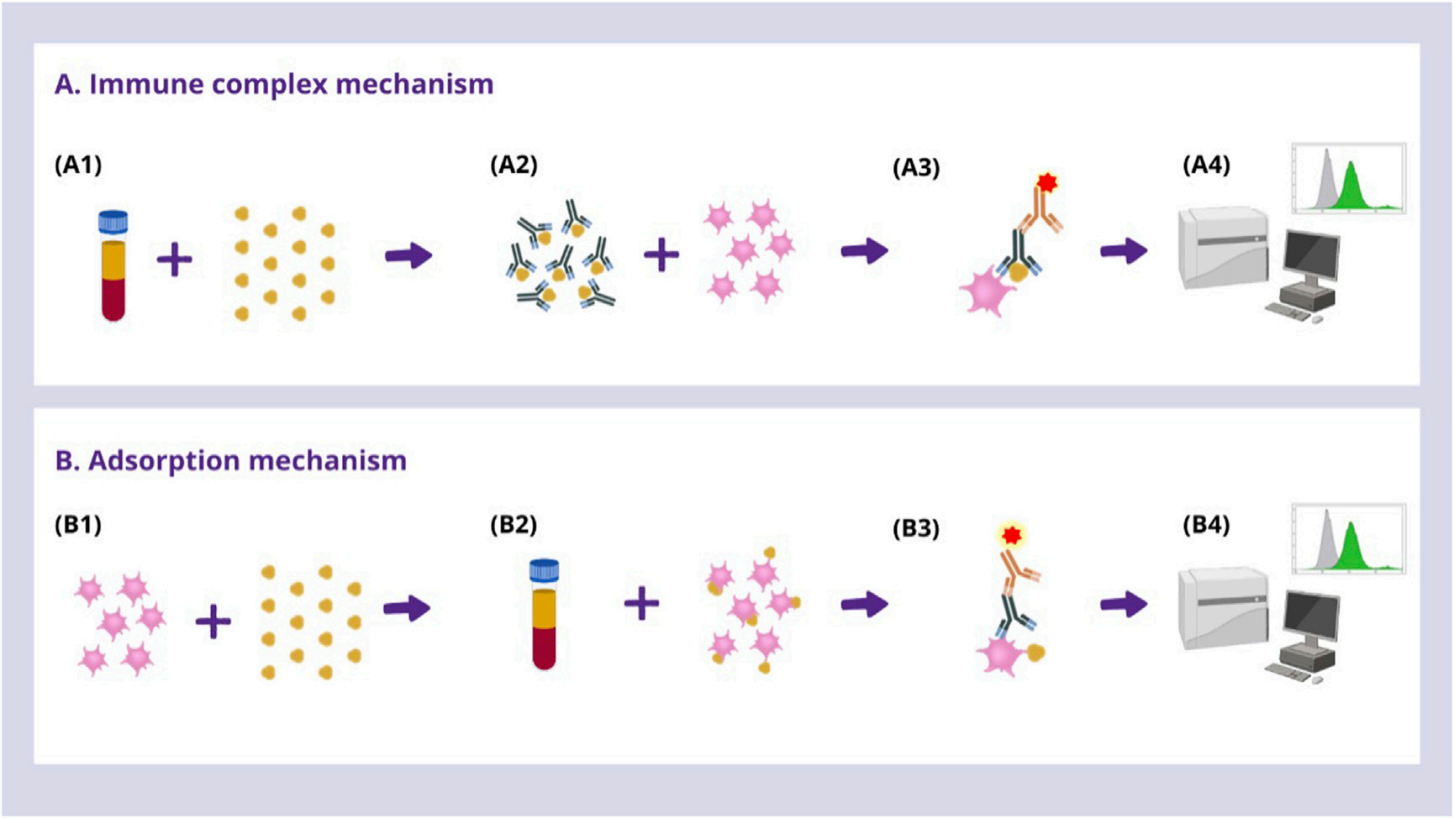
**(A)** First, an immune complex mechanism was assessed. **(B)** In samples with negative results, an adsorption drug-induced cytopenia mechanism was investigated by pre-incubation of donor platelets with each drug prior to performing the indirect test. **(A1)** Pre-incubation between the patient’s serum and the drug to induce in vitro formation of immune complexes. **(A2)** Cross-matching between the patient’s serum -where immune complexes are presumed to have formed- and with freshly untipped healthy O-donor platelets to sensitise them. **(B1)** Pre-incubation between freshly untipped healthy O-donor platelets and the drug to induce its adsorption to the platelet membrane. **(B2)** Cross-matching between the drug-adsorbed platelets and the patient’s serum. **(A3, B3)** Indirect immunoglobulin test by immunofluorescence is performed using three different Fragment Affinity‐Purified antibodies F(ab’)2 goat anti‐human globulins (IgG+IgM, IgG and IgM), labelled with fluorescein isothiocyanate (FITC) (Jackson ImmunoResearch, Newmarket, UK). **(A4, B4)** The sample is analysed by flow cytometry to investigate the presence of DDAbs coating the platelet membrane.

Laboratory testing was conducted in several phases, following standardised protocols (Arnold et al., 2015). Platelets were isolated from healthy donors and then incubated with the patient’s serum and the drug. Detection of DDAbs was then performed by flow cytometry using fluorescence-labelled goat anti-human IgG and IgM antibodies. To ensure that the thrombocytopenia was drug-dependent, the assay was performed in parallel in the absence of the drug and washed with drug-free buffer. In addition, the test was considered positive only when antibody binding to platelets was observed in the presence of the drug and not in its absence, with a mean fluorescence intensity at least 2 standard deviations above that of the negative control (Arnold et al., 2015). OXL was tested at 0.1 mg/mL and 0.2 mg/mL. See Figure 1 for further details on the technique.

### 2.4 Therapeutic management of II-HSRs

Patients with suspected or confirmed II-HSRs were not re-exposed to OXL, except in selected cases who satisfied *all of the following criteria:* (1) absolute oncological indication to continue treatment; (2) equally effective therapeutic alternatives unavailable; (3) non-life-threatening index reaction; (4) rapid recovery from the index reaction; (5) the benefit of continuing treatment outweighs the risks; (6) informed written consent for re-exposure. Although no formal laboratory thresholds were defined to contraindicate re-exposure, biomarkers suggestive of severe systemic involvement (e.g., organ dysfunction, coagulopathy) were considered during risk assessment on a case-by-case basis.

Re-exposure was conducted at DHDC by a highly specialised team, with continuous monitoring and serial analytical controls for 7 days post-OXL infusion. For detailed patient requirements, refer to Section B in the Supplementary Material. We followed standard OXL infusion protocols as per local guidelines, except for patients with concomitant I-HSRs confirmed by positive OXL ST results, who were re-exposed to OXL by using our one-bag RDD protocol (Vázquez-Revuelta et al., 2022). See Section C in the Supplementary Material for further data. All candidate patients were discussed in the allergy multidisciplinary meeting involving all direct care staff—oncologists, nurses, intensivists, and pharmacists. Patients were directly involved in decision-making and signed informed consent.

Management of breakthrough reactions (BTRs) was highly individualised, guided by clinical manifestations, severity, and prior patient history. During the acute phase of BTRs, hypersensitivity biomarkers — including tryptase and IL-6 — were analysed to better characterise the underlying mechanism (see Table 5 for further details). Systemic corticosteroids (such as intravenous boluses of dexamethasone or methylprednisolone at 1–1.5 mg/kg/day for more severe reactions) were prioritised for non-mild cases, considering the presumed immune-mediated aetiology. Haematological support, including platelet, red blood cell, or plasma transfusions, was provided for severe cytopenias or increased bleeding risk (e.g., <20,000/µL in asymptomatic patients, <50,000/µL if bleeding was present). Additional supportive care measures, such as antipyretics or intravenous fluids, were used as clinically indicated. BTRs differing from II-HSRs, such as I-HSRs, were treated according to their specific guidelines (Pagani et al., 2022; Madrigal-Burgaleta et al., 2022; Vázquez-Revuelta et al., 2022). Due to the limited awareness of II-HSRs and their complexities, a dedicated management pathway with multidepartmental SOPs was established to ensure rapid access to standardised care for reactive patients.

## 3 Results

### 3.1 Incidence and characteristics of II-HSRs to OXL

**FIGURE 2:**
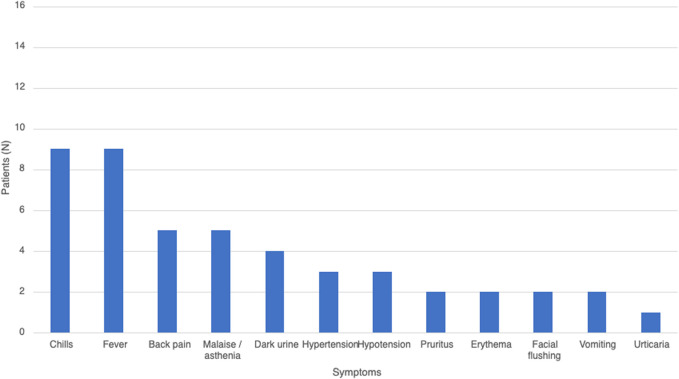
Most frequent symptoms at index reaction.

1,434 patients in our centre received OXL-containing regimes between June 2019 and April 2024. During this period, 12% (169/1,434) were referred to our DHDC after a reaction to OXL, and 16 patients were ultimately diagnosed with II-HSRs (25% women, mean age 63): one in 2019, five in 2021, three in 2022, five in 2023 and two in 2024, resulting in a prevalence for II-HSRs of 9.5% among reactive patients. All patients received OXL for gastrointestinal neoplasms, 88% (14/16) as a retreatment—meaning they had previously received it for another disease setting. See Section D in the Supplementary Material for details on tumour features. At the time of the index reaction, the mean cumulative number of cycles was 20 (range 13–35) in the overall cohort. In the retreatment population, this meant that the index reaction happened on cycle 9 (range 1–18) of the new treatment in average. All patients experienced symptoms during the OXL infusion or within the next hour. Most patients (94%, 15/16) reported between one and 3 previous reactions that had been overlooked. Table 2 summarises the main patient characteristics, clinical presentation of II-HSRs, final diagnosis and subsequent outcomes. The most frequently reported symptoms were chills and fever (both 56%), followed by back pain (31%), malaise/ severe asthenia (31%), dark urine (25%), hypertension (19%) and hypotension (19%). Less common symptoms included vomiting, pruritus/erythema (not hives), flushing, bleeding, abdominal pain, cough, dyspnoea and oxygen desaturation, each reported in two or fewer patients. See Figure 2.

**TABLE 2:** Characteristics of II-HSRS to OXL.

Case No.	Age, Sex	Cum of OXL cycles (n)	Retreatment (N cycles at current retreatment)	Symptoms (time of onset)	Drop of Hb (g/L)	Hae-molysis criteria	DAT	Thrombo-cytopenia	Plt count (N x10^9^/L) (% drop)	DDAbs test (drug)	Organ injury	Final diagnosis	Outcome
1	64, F	19	Yes (8)	Severe asthenia, chills, fever 38.2°C, tachycardia (<1h)	98 → 78	Yes	Positive (IgG)	Yes	574 → 129 (78%)	Positive (OXL)	No	ES	Definite discontinuation
2	59, F	34	Yes (11)	Back pain, joints pain, hypertension (<1h)	130 → 112	No	Positive (IgG)	Yes	136 → 21 (85%)	Positive (OXL, leucovorin)	No	AIT	Definite discontinuation
3	62, F	15	Yes (4)	Back pain, fever 38.5°C, chills, hypertension, vomiting (<1h)	135 → 116	Yes	Positive (IgG, Cd3)	Yes	201 → 120 (40%)	Positive (OXL)	No	AIT	Re-exposure
4	51, M	22	Yes (3)	Generalised urticaria, ear and oral pruritus (<1h) Fever 37.6°C (5h)	142 → 135	No	Negative	Yes	173 → 56 (67%)	Positive (OXL)	No	AIT	Re-exposure
5	65, M	19	No	Chills, fever 39.1°C (0h)	No	No	Negative	Yes	132 → 76 (33%)	Positive (OXL)	No	AIT	Definite discontinuation
6	53, M	13	Yes (2)	Cough, flushing, palms pruritus, erythema (<1h) Fever 39.2°C, chills (9h) Asthenia, hypotension (24h)	110 → 95	Yes	Negative	Yes	232 → 105 (55%)	NA	No	ES	Re-exposure
7	66, M	24	Yes (5)	Chest pain, chills (<1h) Fever 38°C, dark urine (2h)	134 → 132	Yes	Positive	Yes	225 → 144 (36%)	NA	Yes (kidney)	ES	Definite discontinuation
8	64, M	13	Yes (3)	Back pain (<1h) Dark urine (4h)	160 → 146	Yes	Negative	Yes	141 → 24 (83%)	Negative	Yes (kidney)	DITMA	Definite discontinuation
9	63, F	18	Yes (8)	Back pain, fever 38°C, malaise, vomiting, hypotension (<1h)	136 → 119	Yes	Negative	Yes	178 → 37 (80%)	NA	No	ES	Definite discontinuation
10	73, M	15	Yes (2)	Syncope, hypotension, chills, erythema, abdominal pain (<1h)	125 → 109	No	Negative	Yes	146 → 13 (91%)	NA	No	AIT	Definite discontinuation
11	68, M	21	No	Flushing, dyspnoea, diaphoresis, hypertension, oxygen desaturation (9h) (<1h) Malaise, oliguria dark urine (2h)	92 → 70	No	Positive (IgG&Cd3)	Yes	167 → 60 (64%)	NA	Yes (liver, kidney)	AIT	Definite discontinuation
12	60, M	20	Yes (1)	None	98 → 111	No	Positive (IgG)	Yes	389 → 272 (30%)	NA	No	AIT	Re-exposure
13	65, M	19	Yes (11)	Chills, fever 38°C (<1h)	128 → 106	NA	NA	Yes	168 → 78 (54%)	NA	No	AIT	Definite discontinuation
14	68, M	19	Yes (7)	Flushing, epiphora, chills, diarrhoea, rigors (<1h) Asthenia for 12h Dark urine for 48h	111 → 108	Yes	Positive (IgG)	Yes	202 → 120 (41%)	NA	Yes (liver)	ES	Definite discontinuation
15	61, M	35	Yes (18)	Gingivorrhoea, petechiae, epistaxis (<1h)	127 → 105	No	Positive (IgG)	Yes	170 → 0 (100%)	Positive (OXL, leucovorin and ondansetron)	No	AIT	Definite discontinuation
16	64, M	19	Yes (9)	Back pain, chills, fever 38.6°C (<1h)	116 → 95	No	Negative	Yes	249 → 152 (39%)	NA	No	ES	Re-exposure

Hb: haemoglobin, DAT: direct antiglobulin test, Plt: platelets, DDAbs: drug-dependent antibodies, Ig: immunoglobulin, AIT: acute immune thrombocytopenia, IHA: immune haemolytic anaemia, ES: evans syndrome, DITMA: drug-induced thrombotic microangiopathy, NA: not available, OXL: oxaliplatin.

### 3.2 Diagnostic findings

All patients exhibited acute thrombocytopenia within hours of the reaction, with a mean platelet count decrease of 61% (range 30%–100%) from baseline pre-chemotherapy levels. Thrombocytopenia was detected as early as 1 h post-reaction, with the greatest decline typically observed between 24 and 48 h after infusion. A haemolytic pattern was observed in 44% (7/16) of all patients, with a positive direct antiglobulin test (DAT) in 50% (8/16); although only four of these patients (4/8) met the criteria of haemolysis. See Table 2 for more details on II-HSRs findings. One patient (case 16) experienced acute neutropenia with a 95% drop from baseline. Two patients developed renal damage, with one also exhibiting liver dysfunction. Diagnoses included AIT in 56% (9/16) of patients, ES in 38% (6/16), and DITMA in 6% (1/6). See Table 3 for tryptase and IL-6 results at baseline and during index reaction.

**TABLE 3:** Analysis of Hypersensitivity biomarkers on index II-HSRs.

Case no.	Tryptase (µg/L)		Interleucin-6 (ng/L)	
	Baseline	Index Reaction	Baseline	IL-6 (ng/L)
1	4.6	NA	4.4	NA
2^a^	2	2	<3.5	217
3	6	6.2	5	279
4^b^	4	NA	<3.5	21.7
5^a^	6.2	8.5	<3.5	5,477
6^b^	5.3	12.4	4	55.4
7^a^	6	9.8	<3.5	1,276
8^b^	14.9	12.6	6	51.6
9^b^	14.6	16.1	6	1,000
10^a^	2.5	3.9	6.4	7,391
11	NA	NA	NA	NA
12	4.3	NA	4.5	492
13	23	20	18.2	990
14	8.3	11.3	6	710
15	8	NA	5.6	86.7
16	33.1	20.6	29.9	1,330

^a^ At the moment of the Type-II, index reaction, these patients were receiving OXL, under RDD, protocol due to a previous Type I reaction (with positive ST).

^b^ At the moment of the Type–II, index reaction, these patients presented with mixed symptoms and skin testing resulted positive, so a mixed hypersensitivity (type I and II) was diagnosed. NA: not available, OXL: oxaliplatin, RDD: rapid drug desensitisation, ST: skin testing.

### 3.3 Detection of DDAbs by flow cytometry

Flow cytometry was performed on serum from 8 of the 16 patients as this technique was not previously available. DDAbs specific for OXL were observed on serum from six patients (cases 1–5 and 15), with the only negative results being serum from case 8. In case 16, because the indirect test was positive prior to incubation with drug, we were unable to complete the assay. In addition to OXL, DDAbs specific for other drugs were found in the sera of two patients: case 2 had DDAbs specific for leucovorin and case 15 had DDAbs specific for both ondansetron and leucovorin.

### 3.4 Therapeutic management of II-HSR

Five patients (cases 3, 4, 6, 12, and 16) were re-exposed to OXL between one and seven additional times. Re-exposure was considered only for patients who met strict safety criteria, as described in the Methods section. The remaining patients discontinued OXL due to life-threatening reactions, organ involvement, disease progression, or lack of consent. Cases 3 and 4 had confirmed DDAbs for OXL. Cases 4 and 6 received OXL under a RDD protocol due to a type I-IgE-mediated allergy (confirmed by positive ST results). All patients developed new-onset II-HSRs at the time of re-exposure, which responded adequately to treatment. Case 12 tolerated the second re-exposure poorly, with 45% basal platelet decrease, severe back pain, vomiting, severe headache and diaphoresis, but no haemodynamic instability. Despite good response to symptomatic treatment, OXL was permanently discontinued in this patient. Cases 3, 4, 6 and 16 were able to continue treatment with symptomatic treatment and close monitoring until disease progression without major complications, despite reactions to re-exposure. Details of OXL re-exposures and BTRs are presented in Table 4.

**TABLE 4:** OXL re-exposure outcomes and characteristics of breakthrough reactions.

Case	Initial II-HSR subtype	RE protocol	RE	BTR (symptoms)	BTR type	Lab results within first 48h^a^	Treatment	Reason for OXL withdrawal
3	ES	Regular infusion + previous IV dexketoprofen	RE-1	No	Type II (ES)	Thrombocytopenia (141 > 93, 34% drop) Haemolysis	None	*Cumulative toxicity*
			RE-2	Yes (chest tightness)	Type II (ES)	Thrombocytopenia (256 > 148, 42% drop) Haemolysis	1 g IV paracetamol	
			RE-3	No	Type II (ES)	Thrombocytopenia (148 > 124, 16% drop) Haemolysis	None	
			RE-4	Yes (left arm pain)	Type II (ES)	Thrombocytopenia (200 > 120, 40% drop) Haemolysis	1 g IV paracetamol	
			RE-5	Yes (lower back pain, both arms-pain)	Type II (ES)	Thrombocytopenia (166 > 92, 45% drop) Haemolysis	1 g IV paracetamol	
			RE-6	Yes (lower back pain, chest tightness, arm-pain)	Type II (ES)	Thrombocytopenia (224 > 114, 49% drop) Haemolysis	1 g IV paracetamol	
			RE-7	No	Type II (ES)	Thrombocytopenia (214 > 114, 47% drop) Haemolysis	None	
4	AIT	One-bag RDD	RE-1	Yes (urticaria, pink urine)	Mixed (AIT + IgE)	Thrombocytopenia (126 > 61, 52% drop)	300 mg IV hydrocortisone (single dose), 5 mg IV dexclorpheniramine	Disease progression
			RE-2	Yes (urticaria)	Mixed (AIT + IgE)	Thrombocytopenia (107 > 44, 59% drop)	150 mg IV hydrocortisone (single dose)	
			RE-3	Yes (urticaria)	Mixed (ES + IgE)	Thrombocytopenia (125 > 48, 61% drop) Haemolysis DAT+	5 mg IV dexchlorpheniramine	
			RE-4	Yes (face and chest erythema)	Mixed (ES + IgE)	Thrombocytopenia (226 > 76, 66% drop) Haemolysis	5 mg IV dexchlorpheniramine, 200 IV hydrocortisone (single dose)	
			RE-5	Yes (facial urticaria)	Mixed (ES + IgE)	Thrombocytopenia (126 > 59, 53% drop) Haemolysis	None	
			RE-6	Yes (facial erythema)	Mixed (ES + IgE)	Thrombocytopenia (132 > 58, 56% drop) Haemolysis	None	
6	ES	One-bag RDD	RE-1	Yes (headache, back pain, fever 38.4°C)	Type II (ES)	Thrombocytopenia (253 > 159, 37% drop) Haemolysis DAT-	50 mg IV dexketoprofen	Disease progression
			RE-2	Yes (fever 38.4°C after 4h)	Type II (ES)	Thrombocytopenia (255 > 194, 24% drop) Haemolysis DAT+	100 mg IV hydrocortisone (single dose)	
			RE-3	Yes (back pain, dark urine, nausea)	Type II (IHA)	Haemolysis DAT+	1 g IV paracetamol, 100 mg IV hydrocortisone, 8 mg IV ondansetron	
			RE-4	Yes (back pain, nausea, vomiting during infusion)	Type II (ES)	Thrombocytopenia (236 > 150, 36% drop) Haemolysis	10 mg IV metoclopramide, 100 mg IV hydrocortisone (single dose), 8 mg IV ondansetron	
			RE-5	Yes (mild headache after 8h)	Type II (ES)	Thrombocytopenia (262 > 196, 25% drop) Haemolysis	1 g IV paracetamol, 100 mg IV hydrocortisone, 8 mg IV ondansetron	
12	AIT	Regular infusion	RE-1	No	Type II (AIT)	Thrombocytopenia (514 > 408, 20% drop)	none	Severe symptoms on BTR
			RE-2	Yes (severe back pain, chills, severe headache, vomiting, fever, diaphoresis)	Type II (AIT)	Thrombocytopenia (528 > 296, 44% drop)	50 mg IV dexketoprofen, 25 mg IM pethidine	
16	ES	Regular infusion	RE-1	Yes (moderate back-pain)	Type II (ES)	Thrombocytopenia (224 > 107, 52% drop of 52%) Neutropenia (5.8 > 1.8, 69% drop)	50 mg IV dexketoprofen	Disease progression
		Regular infusion + previous IV dexketoprofen	RE-2	Yes (mild chills)		Thrombocytopenia (195 > 91, 53% drop) Neutropenia (3.4 > 0.09, 98% drop)	None	
			RE-3	Yes (chills)		Thrombocytopenia (137 > 34, 76% drop) Neutropenia (5.5 > 0.9, 84% drop)	None	

^a^ Platelets and neutrophils in Nx10′9/L.

II-HSR: type II, hypersensitivity reaction, RE: re-exposure, RDD: rapid drug desensitisation, BTR: breakthrough reaction, OXL: oxaliplatin, DAT: direct antiglobulin test, AIT: acute immune thrombocytopenia, IHA: immune haemolytic anaemia, ES: evans syndrome.

## 4 Discussion

Over a 5-year period, we identified 16 cases of OXL induced II-HSRs in patients treated at our DHDC. All patients presented with immediate-onset symptoms during or shortly after oxaliplatin infusion. Thrombocytopenia was observed in 100% of cases, and 44% showed evidence of haemolysis. DDAbs specific for OXL were detected in 75% (6/8) of the tested patients. Five patients were safely re-exposed under strict protocols, although all developed new II-HSRs.

These findings suggest that II-HSRs to OXL may be under-recognised, often present with immediate-onset symptoms, and can mimic type I reactions or CRRs. The identification of DDAbs supports a type II mechanism in many cases. Importantly, our experience shows that re-exposure to OXL may be feasible in selected patients within a highly controlled setting, with appropriate safety measures.

During this period, we observed fluctuations in incidence, ranging from one case in 2019 to five cases in both 2021 and 2023. Notably, in the first 4 months of 2024, two cases have already been diagnosed, suggesting a potential rise in incidence if this trend continues throughout the year. This apparent increase may be explained by a rise in clinical awareness and the benefits of a multidisciplinary approach that improves diagnostic accuracy and patient identification and management. Despite this, the incidence may be higher as some OXL reactions are not referred to allergy departments for various reasons. The incidence of II-HSRs to OXL remains uncertain due to the limited number of reports available (Bencardino et al., 2025).

Bencardino et al. (2025) aggregated data from 61 published cases, finding a median patient age of 60 years, with 58% being female. Most cases occurred after a mean of 16 OXL cycles, thrombocytopenia was observed in 88.5% of patients, and AIT was the most frequently diagnosed subtype, which aligns with our results. In our cohort, most cases were men, which differs from previous studies showing a higher prevalence in women (Bencardino et al., 2025). Patients experienced II-HSRs after a mean of 20 cumulative OXL cycles, which is slightly higher than previous reports (Bencardino et al., 2025). In our cohort, all patients presented with thrombocytopenia, being the most common cytopenia in OXL-induced II-HSRs.

Although II-HSRs are typically considered non-immediate, all patients in our study experienced immediate symptoms during OXL infusion or within 1 hour. Some of these clinical manifestations, such as pruritus, erythema or hypotension, may be mistaken for I-HSRs. However, the presence of atypical symptoms such as fever, chills and back pain may play a key role in the early diagnosis of II-HSRs. On the other hand, these atypical symptoms may also be confused or even overlap with other drug reactions such as cytokine release syndrome (CRS) or cytokine release reactions (CRRs), which are often associated with thermal changes and significant constitutional and neuromuscular symptoms (Silver et al., 2020; Jakubovic et al., 2021; Jakubovic et al., 2025). CRS and CRR are associated with acute elevation of serum cytokines, with interleukin-6 (IL-6) being one of the most extensively studied biomarkers in this context (Silver et al., 2020; Jakubovic et al., 2021; Jakubovic et al., 2025). In our population, II-HSRs showed a significant increase in IL-6 (as a biomarker of cytokine release in the context of II-HSRs), but not in serum tryptase (a biomarker of mast cell degranulation typical of I-HSRs). Two patients (cases 6 and 7) showed a dynamic increase in tryptase, but this was associated with mixed reactions with IgE-mediated symptoms and positive skin tests. Notably, pruritus and urticaria (typically classified as type I symptoms) were observed only in patients with concomitant I-HSRs, confirmed by positive skin tests. In contrast, none of the patients without type I symptoms had positive skin tests. This supports the notion that type II hypersensitivity is unlikely to induce type I symptoms such as urticaria and pruritus. It is interesting to note that the highest dynamic increases in IL-6 were not always associated with the most severe reactions. Furthermore, significant variations in baseline IL-6 levels were observed between treatment cycles, necessitating a baseline request prior to each procedure (Tables 3, 5). This is of particular importance when considering the overlap between clinical and biomarker features of CRRs and II-HSRs, which can lead to misdiagnosis and potentially life-threatening consequences. We should bear in mind that CRS is not a single condition, but a constellation of symptoms triggered by the release of pro-inflammatory cytokines through several different mechanisms (Jimenez-Rodriguez et al., 2024). Based on our data and findings from previous authors, pro-inflammatory cytokine release can be present in II-HSRs to OXL and mimic a CRS. It is crucial to include II-HSRs in the differential diagnosis when attributing a reaction to a supposed CRR. In addition to measuring IL-6 levels, we recommend early and repeated laboratory workup in all patients presenting with CRR-like symptoms after OXL infusion. This should include, among others, serial complete blood counts, haemolysis parameters, and markers of organ dysfunction (see Section A in Supplementary Material).

**TABLE 5:** Analysis of Hypersensitivity interleukin-6 on breakthrough reactions during OXL re-exposure.

Case no.	RE	Interleucin-6 levels (ng/L)						
		basal	0h	2h	8h	20h	48h	d5
3	RE-1	NA	NA	250	NA	38.7	9.7	NA
	RE-2	9.5	NA	NA	12.8	10.5	12.6	11.5
	RE-3	22.5	NA	71	6.3	4.2	NA	NA
	RE-4	10.7	50.7	105	NA	4.4	16.3	14.1
	RE-5	50.9	NA	77	19.7	10.7	24.7	12.7
	RE-6	205	NA	92	16.3	8.9	18.5	13.2
	RE-7	66.1	NA	164	27.2	NA	19.5	13.3
4	RE-1	<3.5	393	174	7	3.8	<3.5	NA
	RE-2	NA	NA	NA	NA	14.3	24.9	8.7
	RE-3	NA	NA	479	15.5	15.7	<3.5	NA
	RE-4	3.9	NA	1,525	19.7	5.6	12	6.8
	RE-5	5.9	NA	1,873	12.7	<3.5	<3.5	5.2
	RE-6	4.5	NA	1,020	12.1	4.7	6.8	6.2
6	RE-1	NA	NA	NA	118	55.4	11.1	NA
	RE-2	4.7	NA	27.6	35.6	125	NA	NA
	RE-3	NA	NA	112	20.6	NA	NA	NA
	RE-4	NA	NA	NA	29	20.9	8.3	NA
	RE-5	NA	NA	NA	NA	18.3	NA	NA
12	RE-1	33.8	NA	32	10.4	NA	NA	NA
	RE-2	NA	NA	NA	NA	NA	NA	NA
16	RE-1	29.9	3,242	249	NA	4.9	28	NA
	RE-2	NA	440	87.9	NA	6.8	8.9	31.5
	RE-3	NA	NA	129	NA	NA	24.5	NA

RE: re-exposure, NA: not available.

Some studies suggest that haemolysis could explain back pain (Desrame et al., 1999). In our series, four of the five patients with lower back pain (cases 1, 2, 3, 8 and 9) had haemolysis, but one patient (case 2) did not, suggesting that other factors may be involved.

All patients experienced a sudden drop in their platelet count, with an average decrease of 61% within 24–48 h post-OXL infusion, in line with an immune mechanism rather than myelosuppression, which typically occurs 7–10 days post-infusion. We observed that patients with a high baseline platelet count could maintain a count of over 150,000 platelets (the minimum threshold to be considered thrombocytopenia) even after a 50% reduction in baseline. This observation underlines the importance of assessing cytopenias both by absolute counts and percentage reduction from baseline, as patients with high baseline platelet counts might not meet the criteria for thrombocytopenia despite significant drops. Diagnostic scores for cytopenias that use percentage decreases from baseline, like that used for heparin-induced thrombocytopenia, could be beneficial for managing II-HSRs to OXL (Favaloro et al., 2017).

Bencardino *et al.* reported a mortality rate of 6.6% (Bencardino et al., 2025). We believe the high mortality rates for II-HSRs reported in the literature may be influenced by publication bias, as severe cases—often diagnosed late and managed by multiple non-expert teams—are more likely to be documented as case reports due to their perceived novelty. Most of our patients (94%) had experienced previously overlooked reactions, suggesting a broader, underreported spectrum of severity. Outcomes in our cohort were more favourable, with all patients fully recovering without intensive treatment or prolonged hospitalisation. This suggests that morbidity and mortality may be lower with heightened awareness, early detection, multidepartmental SOPs, and timely access to appropriate care.

Flow cytometry confirmed the presence of OXL-specific DDAbs in 6 of the 7 cases with immediate thrombocytopenia and compatible symptoms, reinforcing its utility in diagnosing II-HSRs. The negative result in case 8 could be attributed to the sample being taken 3 years post-index reaction, beyond the optimal detection window of 3 weeks before sensitivity decreases (Arnold et al., 2013). One important limitation of our study is that flow cytometry could only be performed in 8 patients, as this technique became available midway through the project. As a result, some early cases could not be retrospectively analysed using this method. The presence of DDAbs to more than one drug in two patients (cases 2 and 15) highlights that patients who develop DDAbs to OXL can eventually produce DDAbs to other concomitant drugs. Recently, Curtis BR et al. (2018) conducted a retrospective study on serum from 30 patients who, in the context of OXL-induced AIT, were found to have OXL-dependent anti-platelet antibodies. In this analysis, the presence of DDAbs specific to other medications included in their treatment regimens was evaluated. Fourteen (47%) of the 30 serum samples showed 34 DDAbs specific for other drugs: leucovorin (12), irinotecan (2), dexamethasone (7), ondansetron (7), diphenhydramine (2) and palonsetron (4). This recent investigation suggests that patients who develop OXL-dependent DDAbs may have a significant tendency to produce DDAbs specific to other drugs they have been exposed to (Curtis BR. et al., 2018). However, the study showed no data on re-exposure or spontaneous tolerance to those other drugs. In our study, cases 2 and 15 continued to receive leucovorin and ondansetron uneventfully despite the presence of DDAbs, which raises doubts about the clinical relevance of double sensitizations. These results emphasise the need for controls in future studies to determine the prevalence of DDAbs in both healthy individuals and unreactive patients exposed to the same cumulative dose of OXL. Collaboration with laboratories is essential to confirm the diagnosis with appropriate techniques.

Currently, there is no specific treatment for II-HSRs (Jardim et al., 2012). Allergic cytopenias generally resolve after discontinuation of the causative drug, with corticosteroid efficacy remaining uncertain. Management strategies vary widely, from no intervention to prolonged corticosteroid courses or intravenous immunoglobulin administration (Rousseau et al., 2020; Mansouri Taleghani et al., 2005; Akdeniz et al., 2011). In severe cases with major bleeding or organ damage, haematological support may be necessary, including transfusions and, in some instances, haemodialysis for patients with renal involvement (Desrame et al., 1999; Buti et al., 2007; Ulusakarya et al., 2010; Saad et al., 2022). The general recommendation to avoid re-exposure to the drug in suspected II-HSR cases prioritises patient safety, although exceptions may be considered in the absence of therapeutic alternatives, particularly in oncology patients (Knol and Gilles, 2022; Bajwa and Mohammed, 2024). Our results illustrate that a carefully selected subgroup of patients may benefit from drug re-introduction, thus preventing their drug hypersensitivity from interfering with their disease prognosis. However, re-exposure should only be attempted in highly specialised centres, in collaboration with multidisciplinary teams, with standardised pathways and approved multidepartmental SOPs, access to dedicated areas and equipped to manage potential serious complications. These precautions are essential to ensure patient safety, given the morbidity and mortality associated with these reactions.

## 5 Conclusion

OXL-induced II-HSRs are rare but potentially severe and often misdiagnosed due to their clinical overlap with type I and CRRs. Immediate-onset symptoms combined with acute thrombocytopenia and/or haemolysis should raise suspicion. A structured diagnostic approach, including laboratory markers and DDAb testing, is essential for accurate classification and safe management.

Multidisciplinary evaluation—especially involving allergists—is key to recognising these reactions, stratifying risk, and guiding decisions regarding re-exposure. Increasing awareness of II-HSRs and strengthening the role of allergy specialists will support earlier diagnosis, generate high-quality data, and ultimately contribute to the development of evidence-based guidelines that improve patient outcomes.

### 5.1 Clinical implications

This study is the first to systematically investigate the prevalence, clinical characteristics, diagnostic approach, and management of oxaliplatin-induced II-HSRs, highlighting the role of drug-dependent antibodies and the feasibility of controlled re-exposure.

## Data Availability

The original contributions presented in the study are included in the article/Supplementary Material, further inquiries can be directed to the corresponding author.

## Abbreviations

CRC, Colorectal cancer; OXL, oxaliplatin; HSRs, Hypersensitivity reactions; I-HSRs, Type I hypersensitivity reactions; RDD, rapid drug desensitisation; II-HSRs, type II hypersensitivity reactions; IL-6, interleukin-6; AIT, acute immune thrombocytopenia; IHA, immune haemolytic anaemia; ES, Evans syndrome; DITMA, drug-induced thrombotic microangiopathy; DDAbs, DDAbs, drug-dependent reactive autoantibodies; BTRs, breakthrough reactions; DAT, direct antiglobulin test.

## References

[B1] Akdeniz A. Kucukoztas N. Yalcin S. Akcali Z. Altundag O. (2011). Oxaliplatin-induced immune thrombocytopenia in a patient with colon cancer. Am. Surg. 77 (1), E9. 10.1177/000313481107700106 21396291

[B2] Arnold D. M. Kukaswadia S. Nazi I. Esmail A. Dewar L. Smith J. W. (2013). A systematic evaluation of laboratory testing for drug-induced immune thrombocytopenia. J. Thromb. Haemost. 11 (1), 169–176. 10.1111/JTH.12052 23121994 PMC4991941

[B3] Arnold D. M. Curtis B. R. Bakchoul T. Platelet Immunology Scientific Subcommittee of the International Society on Thrombosis and Hemostasis (2015). Recommendations for standardization of laboratory testing for drug-induced immune thrombocytopenia: communication from the SSC of the ISTH. J. Thromb. Haemost. 13 (4), 676–678. 10.1111/JTH.12852 25604471 PMC4854622

[B4] Aster R. H. Bougie D. W. (2007). Drug-induced immune thrombocytopenia. N. Engl. J. Med. 357 (6), 580–587. 10.1056/NEJMRA066469 17687133

[B5] Bajwa S. F. Mohammed R. H. (2024). Type II hypersensitivity reaction. 33085411

[B6] Baretta Z. Falci C. Piva E. Conte P. (2013). Fatal oxaliplatin-induced thrombotic thrombocytopenic purpura: a case report. Clin. Colorectal Cancer 12 (4), 294–296. 10.1016/J.CLCC.2013.09.002 24188688

[B7] Bencardino K. Mauri G. Amatu A. Tosi F. Bonazzina E. Palmeri L. (2025). Oxaliplatin immune-induced syndrome occurs with cumulative administration and rechallenge: single institution series and systematic review study. Clin. Colorectal Cancer 15 (3), 213–221. 10.1016/j.clcc.2016.02.001 26979913

[B8] Buti S. Riccò M. Chiesa M. D. Copercini B. Tomasello G. Brighenti M. (2007). Oxaliplatin-induced hemolytic anemia during adjuvant treatment of a patient with colon cancer: a case report. Anticancer Drugs 18 (3), 297–300. 10.1097/CAD.0B013E3280102F4B 17264762

[B9] Chen V. M. Y. Thrift K. M. Morel-Kopp M. C. Jackson D. Ward C. M. Flower R. L. (2004). An immediate hemolytic reaction induced by repeated administration of oxaliplatin. Transfus. Paris. 44 (6), 838–843. 10.1111/J.1537-2995.2004.03111.X 15157248

[B10] Cobo F. De Celis G. Pereira A. Latorre X. Pujadas J. Albiol S. (2007). Oxaliplatin-induced immune hemolytic anemia: a case report and review of the literature. Anticancer Drugs 18 (8), 973–976. 10.1097/CAD.0B013E3280E9496D 17667605

[B11] Coombs R. R. A. Gell P. G. H. (1968). “Classification of allergic reactions responsible for drug hypersensitivity reactions,” in Clinical aspects of Immunology, 575–596.

[B12] Cunningham D. Starling N. Rao S. Iveson T. Nicolson M. Coxon F. (2008). Capecitabine and oxaliplatin for advanced esophagogastric cancer. N. Engl. J. Med. 358 (1), 36–46. 10.1056/NEJMOA073149 18172173

[B13] Curtis B. R. Kaliszewski J. Marques M. B. Saif M. W. Nabelle L. Blank J. (2006). Immune-mediated thrombocytopenia resulting from sensitivity to oxaliplatin. Am. J. Hematol. 81 (3), 193–198. 10.1002/AJH.20516 16493620

[B14] Curtis S. A. Curtis B. R. Lee A. I. Hendrickson J. E. Lacy J. Podoltsev N. A. (2018a). A patient with oxaliplatin immune-induced syndrome (OIIS) who also developed leucovorin and palonosetron-associated thrombocytopenia. Hematology 23 (7), 429–432. 10.1080/10245332.2017.1419600 29281948

[B15] Curtis B. R. Hsu Y. M. S. Podoltsev N. Lacy J. Curtis S. Samuel M. S. (2018b). Patients treated with oxaliplatin are at risk for thrombocytopenia caused by multiple drug-dependent antibodies. Blood 131 (13), 1486–1489. 10.1182/BLOOD-2017-10-812461 29439950 PMC5877442

[B16] Dahabreh I. Tsoutsos G. Tseligas D. Janinis D. (2006). Hemolytic uremic syndrome following the infusion of oxaliplatin: case report. BMC Clin. Pharmacol. 6, 5. 10.1186/1472-6904-6-5 16968538 PMC1574347

[B17] Demoly P. Adkinson N. F. Brockow K. Castells M. Chiriac A. M. Greenberger P. A. (2014). International Consensus on drug allergy. Allergy 69 (4), 420–437. 10.1111/all.12350 24697291

[B18] Desrame J. Broustet H. Darodes De Tailly P. Girard D. Saissy J. M. (1999). Oxaliplatin-induced haemolytic anaemia. Lancet 354 (9185), 1179–1180. 10.1016/S0140-6736(99)03827-1 10513718

[B19] Dieras V. Bougnoux P. Petit T. Chollet P. Beuzeboc P. Borel C. (2002). Multicentre phase II study of oxaliplatin as a single-agent in cisplatin/carboplatin +/- taxane-pretreated ovarian cancer patients. Ann. Oncol. 13 (2), 258–266. 10.1093/ANNONC/MDF018 11886003

[B20] Doña I. Torres M. J. Celik G. Phillips E. Tanno L. K. Castells M. (2024). Changing patterns in the epidemiology of drug allergy. Allergy 79 (3), 613–628. 10.1111/ALL.15970 38084822

[B21] Erdem G. Dogan M. Demirci N. Zengin N. (2016). Oxaliplatin-induced acute thrombocytopenia. J. Cancer Res. Ther. 12 (2), 509–514. 10.4103/0973-1482.154056 27461601

[B22] Favaloro E. J. McCaughan G. Pasalic L. (2017). Clinical and laboratory diagnosis of heparin induced thrombocytopenia: an update. Pathology 49 (4), 346–355. 10.1016/J.PATHOL.2017.02.005 28446364

[B23] Ferlay J. Soerjomataram I. Dikshit R. Eser S. Mathers C. Rebelo M. (2015). Cancer incidence and mortality worldwide: sources, methods and major patterns in GLOBOCAN 2012. Int. J. Cancer 136 (5), E359–E386. 10.1002/IJC.29210 25220842

[B24] Forcello N. P. Khubchandani S. Patel S. J. Brahaj D. (2015). Oxaliplatin-induced immune-mediated cytopenias: a case report and literature review. J. Oncol. Pharm. Pract. 21 (2), 148–156. 10.1177/1078155213520262 24500808

[B25] Goldberg R. M. Sargent D. J. Morton R. F. Fuchs C. S. Ramanathan R. K. Williamson S. K. (2023). A randomized controlled trial of fluorouracil plus leucovorin, irinotecan, and oxaliplatin combinations in patients with previously untreated metastatic colorectal cancer. J. Clin. Oncol. 41 (19), 3461–3468. 10.1200/JCO.22.02759 37379691

[B26] Ito I. Ito Y. Mizuno M. Suzuki Y. Yasuda K. Ozaki T. (2012). A rare case of acute kidney injury associated with autoimmune hemolytic anemia and thrombocytopenia after long-term usage of oxaliplatin. Clin. Exp. Nephrol. 16 (3), 490–494. 10.1007/S10157-012-0620-8 22450906

[B27] Jakubovic B. D. Sanchez-Sanchez S. Hamadi S. Lynch D. M. Castells M. (2021). Interleukin-6: a novel biomarker for monoclonal antibody and chemotherapy-associated hypersensitivity confirms a cytokine release syndrome phenotype-endotype association. Allergy 76 (5), 1571–1573. 10.1111/ALL.14644 33119137

[B28] Jakubovic B. D. Leticia De L. V. Jimenez-Rodriguez W. Sanchez-Sanchez S. Castells M. (2025). Drug hypersensitivity in the fast lane what clinicians should know about phenotypes. endotypes, biomarkers. 10.1016/j.anai.2020.04.005 32302769

[B29] James E. Podoltsev N. Salehi E. Curtis B. R. Saif M. W. (2009). Oxaliplatin-induced immune thrombocytopenia: another cumulative dose-dependent side effect? Clin. Colorectal Cancer 8 (4), 220–224. 10.3816/CCC.2009.N.037 19822513

[B30] Jardim D. L. Rodrigues C. A. Novis Y. A. S. Rocha V. G. Hoff P. M. (2012). Oxaliplatin-related thrombocytopenia. Ann. Oncol. 23 (8), 1937–1942. 10.1093/ANNONC/MDS074 22534771

[B31] Jimenez-Rodriguez T. W. de las Vecillas L. Labella M. Lynch D. M. Besz K. M. Marquis K. (2024). Differential presentation of hypersensitivity reactions to carboplatin and oxaliplatin: phenotypes, endotypes, and management with desensitization. Allergy 79 (3), 679–689. 10.1111/ALL.15940 37916741

[B32] Jutel M. Agache I. Zemelka-Wiacek M. Akdis M. Chivato T. Del Giacco S. (2023). Nomenclature of allergic diseases and hypersensitivity reactions: adapted to modern needs: an EAACI position paper. Allergy 78 (11), 2851–2874. 10.1111/ALL.15889 37814905

[B33] Knol E. F. Gilles S. (2022). Allergy: type I, II, III, and IV. Handb. Exp. Pharmacol. 268, 31–41. 10.1007/164_2021_510 34255192

[B34] Lucchesi A. Carloni S. Cangini D. Frassineti G. L. Casadei Gardini A. (2013). Acute oxaliplatin-induced thrombotic thrombocytopenic purpura: a case report and results from a cytoflourimetric assay of platelet fibrinogen receptor. J. Clin. Oncol. 31 (16), 2061–2062. 10.1200/JCO.2012.48.3248 23610122

[B35] Madrigal-Burgaleta R. Alvarez-Cuesta E. Madrigal-Burgaleta R. Cuesta-Herranz J. Guzman-Melendez M. A. Maciag M. C. (2022). Standards for practical intravenous rapid drug desensitization and delabeling: a WAO committee statement. World Allergy Organ. J. 15 (6), 100640. 10.1016/J.WAOJOU.2022.100640 35694005 PMC9163606

[B36] Malkhasyan K. Halene S. Lacy J. (2015). Oxaliplatin-related acute disseminated intravascular coagulation syndrome in a patient with metastatic colon cancer. Clin. Colorectal Cancer 14 (1), e9–e12. 10.1016/J.CLCC.2014.09.011 25446051

[B37] Mansouri Taleghani B. Fontana S. Meyer O. Ahrens N. Novak U. Borner M. M. (2005). Oxaliplatin-induced immune pancytopenia. Transfus. Paris. 45 (5), 704–708. 10.1111/J.1537-2995.2005.04373.X 15847658

[B38] Martí-Garrido J. Vázquez-Revuelta P. Lleonart-Bellfill R. Molina-Mata K. Muñoz-Sánchez C. Madrigal-Burgaleta R. (2020). Pilot experience using drug provocation testing for the study of hypersensitivity to chemotherapy and biological agents. J. Investig. Allergol. Clin. Immunol. 31 (2). 10.18176/jiaci.0552 32573462

[B39] Mason J. M. Rees G. J. G. (2011). Oxaliplatin-induced acute thrombocytopenia. J. Oncol. Pharm. Pract. 17 (4), 433–435. 10.1177/1078155210381287 20699331

[B40] Meyerhardt J. A. Mayer R. J. (2005). Systemic therapy for colorectal cancer. N. Engl. J. Med. 352 (5), 476–487. 10.1056/NEJMra040958 15689586

[B41] Nehls O. Oettle H. Hartmann J. T. Hofheinz R. D. Hass H. G. Horger M. S. (2008). Capecitabine plus oxaliplatin as first-line treatment in patients with advanced biliary system adenocarcinoma: a prospective multicentre phase II trial. Br. J. Cancer 98 (2), 309–315. 10.1038/SJ.BJC.6604178 18182984 PMC2361467

[B42] Niu J. Mims M. P. (2012). Oxaliplatin-induced thrombotic thrombocytopenic purpura: case report and literature review. J. Clin. Oncol. 30 (31), e312–e314. 10.1200/JCO.2012.42.5082 22987080

[B43] Pagani M. Bavbek S. Alvarez-Cuesta E. Berna Dursun A. Bonadonna P. Castells M. (2022). Hypersensitivity reactions to chemotherapy: an EAACI position paper. Allergy 77 (2), 388–403. 10.1111/ALL.15113 34587281

[B44] Raymond E. Faivre S. Woynarowski J. M. Chaney S. G. (1998). Oxaliplatin: mechanism of action and antineoplastic activity. Semin. Oncol. 25 (2 Suppl. 5), 4–12. 9609103

[B45] Revuelta P. V. Madrigal-Burgaleta R. Molina G. M. Mata K. M. Serrano C. M. Bellfill R. L. (2023). Assessing our chemotherapy and biologics drug desensitization center. J. Allergy Clin. Immunol. 151 (2), AB57. 10.1016/j.jaci.2022.12.181

[B46] Rousseau C. Nguyen T. N. Rebibou J. M. Bastie J. N. Audia S. Darut-Jouve A. (2020). Oxaliplatin-induced Evans syndrome: a possible dual mechanism. Clin. Colorectal Cancer 19 (1), 57–60. 10.1016/J.CLCC.2019.11.001 31883972

[B47] Saad R. Hannun A. Temraz S. Finianos A. Zeenny R. M. (2022). Oxaliplatin-induced thrombotic microangiopathy: a case report. J. Med. Case Rep. 16 (1), 110. 10.1186/S13256-022-03309-7 35303936 PMC8933951

[B48] Saif M. W. (2006). Hypersensitivity reactions associated with oxaliplatin. Expert Opin. Drug Saf. 5 (5), 687–694. 10.1517/14740338.5.5.687 16907658

[B49] Saif M. W. Ledbetter L. (2009). Oxaliplatin-mediated autoimmune thrombocytopenia. Clin. Colorectal Cancer 8 (1), 61–62. 10.1016/S1533-0028(11)70349-8 19203900

[B50] Shao Y. Y. Hong R. L. (2008). Fatal thrombocytopenia after oxaliplatin-based chemotherapy. Anticancer Res. 28 (5B), 3115–3117. Available online at: https://ar.iiarjournals.org/content/28/5B/3115 (Accessed February 25, 2024). 19031966

[B51] Silver J. Garcia-Neuer M. Lynch D. M. Pasaoglu G. Sloane D. E. Castells M. (2020). Endophenotyping oxaliplatin hypersensitivity: personalizing desensitization to the atypical platin. J. Allergy Clin. Immunol. Pract. 8, 1668–1680.e2. 10.1016/j.jaip.2020.02.013 32112926

[B52] Stack A. Khanal R. Denlinger C. S. (2021). Oxaliplatin-induced immune thrombocytopenia: a case report and literature review. Clin. Colorectal Cancer 20 (1), e1–e4. 10.1016/J.CLCC.2020.07.007 33012678 PMC7855550

[B53] Suzuki K. Oda H. Sugawara Y. Masuya M. Nakase K. Fujioka M. (2013). Oxaliplatin-induced acute thrombocytopenia: a case report and review of the literature. Intern Med. 52 (5), 611–615. 10.2169/INTERNALMEDICINE.52.8933 23448774

[B54] Teng C. J. Hsieh Y. Y. Chen K. W. Chao T. C. Tzeng C. H. Wang W. S. (2011). Sudden-onset pancytopenia with intracranial hemorrhage after oxaliplatin treatment: a case report and literature review. Jpn. J. Clin. Oncol. 41 (1), 125–129. 10.1093/JJCO/HYQ162 20826449

[B55] Ulusakarya A. Misra S. Haydar M. Habert H. Castagne V. Gumus Y. (2010). Acute renal failure related to oxaliplatin-induced intravascular hemolysis. Med. Oncol. 27 (4), 1425–1426. 10.1007/S12032-009-9263-3 19565364

[B56] Vázquez-Revuelta P. Martí-Garrido J. Molina-Mata K. Lleonart-Bellfill R. Rey-Salido Pharm M. Madrigal-Burgaleta R. (2020). Delabeling patients from chemotherapy and biologics allergy: implementing drug provocation testing. J. Allergy Clin. Immunol. Pract. 10.1016/j.jaip.2020.11.021 33242626

[B57] Vázquez-Revuelta P. Lleonart-Bellfill R. Molina-Mata K. Muñoz-Sánchez C. Rey-Salido M. Madrigal-Burgaleta R. (2022). A pilot experience using a one-bag intravenous rapid desensitization protocol for chemotherapeutics and biologics in a cohort of patients with access to a delabeling pathway. J. Investig. Allergol. Clin. Immunol. 33 (4). 10.18176/JIACI.0860 36168929

[B58] Yang B. C. Castells M. C. (2025). The who, what, where, when, why, and how of drug desensitization. Immunol. Allergy Clin. North Am. 42, 403–420. 10.1016/j.iac.2021.12.004 35469626

