# Supplementary material for: Oxaliplatin-induced type II hypersensitivity in colorectal cancer: a cohort study on clinical presentation, diagnosis, and management
Source: Front Pharmacol. 2025 Aug 29;16:1605690. doi: 10.3389/fphar.2025.1605690 (PMC12426282; doi:10.3389/fphar.2025.1605690)
Supplement: Supplementary file 1 [file DataSheet1.pdf]

Glossary

AIT: Acute Immune thrombocytopenia

aPTT: activated partial thromboplastin time

DAT: Direct Antiglobulin Test

DDAPAb: Drug-dependent antiplatelet antibody

DIC: disseminated intravascular coagulation

DITMA: Drug-Induced Thrombotic Microangiopathy

ES: Evans Syndrome

Hb: haemoglobin

IHA: Immune Haemolytic Anaemia

II-HSR: Type II-hypersensitivity reaction

ITP: Immune thrombocytopenic purpura

MAHA: Microangiopathic Haemolytic Anaemia

OXL: Oxaliplatin

PT: Prothrombin time

RBCs: red blood cells

TMA: Thrombotic Microangiopathy

TTP: Thrombotic thrombocytopenic purpura

WBCs: White Blood Cells

SECTION A. Criteria for Identifying Type-II Hypersensitivity Reactions (II-HSRs) Subtypes

Acute Immune Thrombocytopenia (AIT)

Oxaliplatin (OXL) can induce thrombocytopenia via multiple mechanisms, primarily involving immune pathways[1–3]. The immune-mediated thrombocytopenia characteristic of OXL-induced II-HSRs results from cell lysis by drug-dependent antiplatelet antibodies (DDAPAbs) specific to OXL.

Clinical manifestations

- **Thrombocytopenia**: Defined as a platelet count below the lower limit of reference range, typically 150 x 10^9/L for adults. In severe cases of Immune Haemolytic Anaemia (IHA), counts may drop to as low as 2 x 10^9/L. Some patients may have a baseline platelet count slightly below the lower limit of the reference range, while others may have levels above the upper limit. Note that some patients may have a baseline platelet count slightly below the lower limit of the reference range, while others may have levels above the upper limit. In such cases, a significant decrease from baseline should be considered suspicious, and the count should be repeated after 12-24 hours to confirm a downward trend.
- **Spontaneous bleeding**: Bleeding is a concern among patients with severe thrombocytopenia. While other symptoms generally develop gradually, bleeding can sometimes start suddenly. The risk of spontaneous bleeding is more likely with platelet counts below 20 x 10^9/L, typically manifesting on the skin or mucous membranes as ‘platelet-type bleeding’. Other factors such as platelet dysfunction, coagulation abnormalities, disseminated intravascular coagulation (DIC), or severe liver disease may play an important role in bleeding risk.
- **Petechiae**: Small, flat, red, non-blanching lesions caused by dermal or submucosal haemorrhage. These typically appear on dependent areas of the body such as distal legs in ambulatory patients, sacral region in recumbent patients. Petechiae should be distinguished from vasculitic purpura.
- **Ecchymoses (bruises):** non-tender areas of bleeding into the skin. These are typically multiple, and present with various colours due to extravasated blood (red and purple) and breakdown products of heme pigment (green, orange and yellow). Ecchymotic lesions are known for their small size, multiple occurrence and superficial nature. Notably, these lesions may develop without noticeable trauma and do not spread into deeper tissues.
- **Purpura**: Purpura presents as clusters of coalesced petechiae on the skin ('dry purpura'). In mucosal regions, haemorrhagic vesicles (‘wet purpura’) may indicate a higher risk of severe bleeding.
- **Epistaxis**: Minimal epistaxis, such as bleeding when blowing the nose, is common and often harmless. Ongoing epistaxis predicts an increased risk of more severe bleeding.

- **Severe or critical bleeding:** It is less common than minor events like petechiae and purpura. Patients with platelet counts <20 x 10^9/L are more likely to experience clinically significant bleeding.
- **Asthenia:** Common in immune-mediated thrombocytopenias, although the underlying cause of this symptom remains unclear.
- **Symptoms during infusion**[4–6]**:** Systemic symptoms such as light-headedness, chills, fever, nausea and vomiting may occur during infusion of drugs that can induce thrombocytopenia.

Diagnostic evaluation[1]

OXL-induced AIT is a diagnosis of exclusion made when patients present with a sudden drop in platelet count after exposure to OXL, in the absence of other causes. Therefore, exclusion of alternative causes of thrombocytopenia is a key aspect of the diagnostic process (see *Differential Diagnosis*). *In vitro* detection of DDAPAbs specific for OXL is considered confirmatory. However, this test is not routinely available, and the turnaround time for results can be several days, limiting its usefulness for urgent management.

- **Medical history:** Enquire about a family history of platelet disorders, recent infections, current medications (including over-the-counter drugs and herbal remedies), and underlying conditions such as rheumatologic or liver diseases that may cause thrombocytopenia. It is also important to exclude haemorrhagic symptoms, bruising and petechiae.
- **Physical examination**: Conduct a thorough skin examination to identify signs of skin and mucosal bleeding.
- **Laboratory testing** [7,8]

Full blood count: Other cell lines—white blood cells (WBCs), red blood cells (RBCs)— are usually normal in AIT, unless the immune response affects multiple cell lines, as in Evans syndrome.

Peripheral blood smear (to be interpreted by an experienced clinician or clinical laboratory scientist): used to exclude artefactual thrombocytopenia due to platelet clumping ('pseudothrombocytopenia') and to evaluate morphologic alterations of blood cells that could indicate other causes of thrombocytopenia. Platelet morphology in AIT is typically normal, without abnormalities (e.g. lack of platelet granules or uniformly large or small platelets, which may suggest an inherited platelet disorder). Large platelets may be observed in AIT but are not essential for diagnosis. Schistocytes suggest a microangiopathic process (DIC, DITMA), spherocytes suggest immune-mediated haemolytic anaemia.

Other tests: Prothrombin time (PT) and activated partial thromboplastin time (aPTT) may help investigate potential causes of thrombocytopenia (e.g., liver disease) and bleeding (e.g., vitamin K deficiency), especially in cases with actual bleeding or moderate thrombocytopenia with a bleeding risk. A metabolic

panel and liver function testing (hepatic enzymes, coagulation testing, albumin) is useful to evaluate for occult liver disease. Thrombocytopenia can also be seen in hepatitis C virus (HCV) infection. Testing may be appropriate in outpatients with new thrombocytopenia if it has not been done recently.

Detection of DDAPAbs: Demonstrating DDAPAbs *in vitro* is essential for confirming the drug's causative role in the thrombocytopenia, especially when distinguishing between multiple suspected drugs. Common laboratory tests for detecting DDAPAbs include indirect platelet antibody tests (for identifying platelet-specific antibodies in serum or plasma) and direct platelet antibody tests (for detecting autoantibodies on patient platelets) [7]. Methods for detecting DDAPAbs include enzyme immunoassays, flow cytometry, platelet suspension immunofluorescence, radio-labelled antiglobulin-based assays, and glycoprotein-specific assays. Flow cytometry, which uses fluorescently labelled antiglobulins, offers greater sensitivity, quantitative data, and the ability to identify multiple antibody types simultaneously[8]. However, these tests are not widely available in many hospital laboratories.

Differential diagnosis[3,8]

The main differential diagnosis for OXL-induced AIT includes OXL-related thrombocytopenia secondary to non-immunological causes, such as myelosuppression (the most common cause) and splenic sequestration. A thorough evaluation of the patient's medical history is essential, with particular focus on the timing of OXL exposure and platelet recovery after drug discontinuation. Clinical symptoms and laboratory findings are crucial for excluding other causes of acute thrombocytopenia, especially non-immune aetiologies. **Table 1** highlights the critical differences between immune and non-immune OXL-related thrombocytopenia.

Other differential diagnoses to consider include immune-mediated thrombocytopenia (unrelated to OXL), infections, and disseminated intravascular coagulation (DIC).

a) **OXL-related non-immune thrombocytopenia:**
   a. **Myelosupression**: characterised by a concurrent reduction in platelets, WBCs, and RBCs, distinguishing it from autoimmune thrombocytopenia (AIT), where only platelets are primarily affected.
   b. **Splenic sequestration:** marked by a persistent drop in platelet count in the presence of splenomegaly.

b) **Immune-mediated thrombocytopenia (unrelated to OXL):**
   a. **Immune thrombocytopenic purpura (ITP):** The diagnosis of ITP is one of exclusion, defined as isolated thrombocytopenia without anaemia or leukopenia and in the absence of other causes. ITP is an autoimmune phenomenon of unknown aetiology caused by autoantibody (typically

IgG) against platelet antigens, most commonly platelet membrane glycoproteins such as GPIIb/IIIa. In OXL-induced AIT, the autoantibodies are not activated in the absence of the drug. Clinically, ITP and AIT are often indistinguishable. As with AIT, ITP is characterised by high variability in platelet counts, in contrast to other causes of thrombocytopenia (e.g., liver disease, hereditary thrombocytopenia), where levels are more stable. The clinical history is important to rule out other possible causes of thrombocytopenia, such as the introduction of new drugs and the latency period between drug exposure and thrombocytopenia**.**

b. **Infections:** Various viral (e.g., HIV, hepatitis C) and bacterial infections can cause thrombocytopenia. Differentiation from drug-induced immune thrombocytopenia depends on clinical context and appropriate testing for underlying infections.

TABLE 1. Main differences between immune and non-immune OXL-induced thrombocytopenia

	Immune thrombocytopenia	Non-immune thrombocytopenia	
	**Immune-mediated**	**Myelosuppression**	**Splenic sequestration**
**Time of onset**	Usually after 12 cycles	From 1st cycle onwards	Usually after 18 weeks
**Speed of platelet count reduction**	Acute (hours)	Gradual (≈10-28 days)	Gradual (≈18 weeks)
**Usual platelet count Nadir**	It can be as low as 2×10^9/L	≈ 75×10^9/L	≈ 81×10^9/L
**Mechanism**	OXL-dependent antibodies to platelet antigens	Toxicity to megakaryocytic progenitors	OXL-induced sinusoidal injury leading to portal hypertension
**Clinical presentation**	Sudden and isolated drop in platelet levels with clinical bleeding. *Infusion reactions may occur*	Asymptomatic thrombocytopenia usually with anaemia and neutropenia	Prolonged thrombocytopenia in the context of splenomegaly and other features of portal hypertension
**Diagnostic evaluation**	In vitro detection of OXL dependent antibodies (e.g., flow cytometry)	Bone marrow aspirate not routinely carried out	Detection of splenomegaly in imaging studies
**Management**	Definitive discontinuation of OXL. Consider supportive care, e.g. platelet transfusion, corticosteroids, fluids	Dose reduction or treatment delays. Consider platelet-stimulating agents	Temporary discontinuation of OXL +/- consider partial splenic embolisation

Adapted from Jardim DL, et al. Oxaliplatin-related thrombocytopenia. Annals of Oncology. 2012;23(8):1937-1942.

c. **Disseminated Intravascular coagulation (DIC)**: DIC is a systemic condition characterised by widespread clotting activation, leading to the consumption of platelets and clotting factors. It is typically associated with sepsis, trauma, or cancer and can be differentiated by abnormal clotting tests (e.g., elevated D-dimer, prolonged PT/PTT).

Immune haemolytic anaemia (IHA)[9–11]

Haemolytic anaemia is characterised by the premature destruction of circulating RBCs, leading to a significantly reduced lifespan. This condition can arise from a variety of underlying causes, both inherited and acquired. This condition may stem from a range of underlying factors, both inherited and acquired, with drugs recognised as a significant contributor among the acquired causes.

The two primary mechanisms of drug-induced haemolysis are:

- **Destruction due to oxidant injury**: This occurs in conditions like glucose-6-phosphate dehydrogenase (G6PD) deficiency.
- **Immune-mediated destruction**: This mechanism leads to immune haemolytic anaemia (IHA), where RBCs destruction is primarily antibody-mediated through various antigen-antibody interactions.

Haemolysis can also be classified based on the location of RBC destruction:

- **Intravascular haemolysis:** RBCs are lysed within the circulation, releasing free Hb into the bloodstream. This can manifest clinically as pink urine (from oxyhaemoglobin) or dark brown urine (from methaemoglobin, the oxidised form). Complications include acute kidney injury, DIC, and an elevated risk of thrombosis.
- **Extravascular haemolysis:** RBCs destruction occurs outside the bloodstream, primarily within the spleen, bone marrow, or lymph nodes.

In most cases of IHA, antibody-coated RBCs are phagocytised by reticuloendothelial macrophages in the spleen and liver, leading to extravascular haemolysis. Although the direct antiglobulin test (DAT) is typically positive in IHA, exceptions can occur.

The pattern of haemolysis—intravascular, extravascular, or mixed—has a profound impact on clinical presentation and outcomes. While IHA predominantly involves extravascular haemolysis, severe cases may

exhibit mixed features of both types. Identifying the site of RBCs destruction is essential for diagnosing the underlying cause of haemolysis and guiding appropriate treatment strategies.

Clinical manifestations[12–19]

A thorough history and physical examination can provide valuable clues for diagnosing haemolytic anaemia. Key findings to consider include:

- **Initiation of a new medication:** The use of a drug with known potential to cause haemolysis suggests a possible drug-induced aetiology.
- **Jaundice and dark or red urine:** These findings, often caused by haemoglobinuria, suggest brisk haemolysis that exceeds the reticuloendothelial system's ability to convert haem into storage iron.
- **Signs of intravascular haemolysis:** Dark urine and elevated lactate dehydrogenase (LDH) are indicative of intravascular haemolysis.
- **Systemic symptoms**: Sudden fatigue, weakness, pallor, jaundice, dark urine, abdominal pain, back pain, dyspnoea, and signs of volume depletion without evidence of bleeding are consistent with rapid haemolysis.

It is important to note that the absence of these features does not rule out haemolytic anaemia, as some patients may exhibit minimal or no symptoms.

Diagnosis

IHA must be suspected when patients exhibit acute haemolysis after exposure to a drug in the absence of other causes. Therefore, the initial step in the diagnostic process is to recognise the signs and symptoms of haemolysis. The diagnosis is based on the following findings:

- **Increased LDH, increased indirect bilirubin, and low haptoglobin**: These findings indicate RBCs destruction. Haemolysis results in the release of LDH and Hb from RBCs. Circulating Hb binds to haptoglobin, facilitating haem recycling. Subsequently, Hb undergoes degradation and is converted into bilirubin as part of the metabolic process.
- **Low haptoglobin:** Low levels are indicative of haemolysis, and undetectable levels almost always confirm it. However, elevated haptoglobin does not exclude haemolysis, as haptoglobin is an acute-phase reactant that can be elevated in the context of inflammation.
- **Anaemia:** defined as a decrease in the number of RBCs (measured by haematocrit or Hb content). The presence of anaemia depends on the extent of haemolysis and the bone marrow's ability to compensate by increasing RBC production. Haemolysis stimulates compensatory RBC production via the kidneys,

which increases erythropoietin secretion. This, in turn, stimulates the bone marrow to produce more RBC precursors, leading to an increase in Hb and haematocrit over a few days.

- **Reticulocytosis:** characterised by an increased percentage of reticulocytes in the peripheral blood. This occurs as the bone marrow compensates for reduced Hb levels by increasing erythropoietic activity and releasing reticulocytes into the circulation. However, reticulocytosis is a common response to anaemia but not specific to haemolysis. It may also result from accelerated RBC production due to other causes, such as recent bleeding or the repletion of iron, vitamin B12, folate, copper, or erythropoietin. Patients with iron or vitamin deficiencies or bone marrow issues may not exhibit reticulocytosis despite ongoing haemolysis.

Additional tests can provide critical insights into the specific causes of haemolysis. For instance, findings such as schistocytes or spherocytes on a peripheral blood smear (PBS), free Hb or pink serum, or a newly positive DAT can support the diagnosis. However, the absence of these findings does not rule out haemolysis.

- **Full blood count (FBC):** Most patients with haemolysis exhibit some degree of anaemia, particularly if RBC production is delayed in response to haemolysis. In severe cases where the bone marrow cannot adequately compensate, anaemia may be more pronounced.
- **Peripheral blood smear (PBS):** Examination of the PBS is an invaluable tool for diagnosing and determining the underlying cause of haemolytic anaemia. Smear findings often guide critical treatment strategies, such as the identification of drug-induced thrombotic microangiopathy (DITMA), which may require life-saving interventions.
- **DAT**: Typically positive in cases where red blood cells are coated with Immunoglobulin G (IgG) and/or complement, providing evidence of immune-mediated haemolysis[20].

Evans Syndrome (ES)[3,4,21]

Defined as the co-occurrence of two or more immune-mediated cytopenias, most often anaemia and thrombocytopenia[21,22]. The majority of OXL-associated ES cases manifest as thrombocytopenia and haemolytic anaemia, although cases of drug-induced ES with neutropenia have also been reported[4].

Drug-induced thrombotic microangiopathy (DITMA)

Thrombotic microangiopathy (TMA) is a life-threatening syndrome characterised by the presence of microangiopathic haemolytic anaemia (MAHA), thrombocytopenia, and variable end-organ damage resulting from thrombi in the microcirculation[12]. This condition can have various aetiologies, including

genetic deficiencies, coagulation factor disorders, complement dysregulation, and bacterial infections. In oncology patients, TMA is most commonly secondary to sepsis or disseminated malignancy per se, typically from breast, stomach and colon adenocarcinomas[14]. Several drugs—including quinine, calcineurin inhibitors (CNI) and mammalian target of rapamycin (mTOR) inhibitors—have been identified as causative agents of DITMA, although the underlying mechanisms remain not yet fully understood. Case reports have also described DITMAs in association with antineoplastic agents such as gemcitabine, cisplatin, carboplatin, docetaxel, bortezomib, mitomycin, bleomycin and OXL[14,23].

DITMA should be considered in any patient presenting with an unexplained acute drop in platelet count, haemolysis, and kidney function decline after exposure to a relevant drug.

Clinical manifestations[12,14,17,24]

The classic clinical features of immune-mediated DITMA are illustrated by the experience with quinine-induced DITMA[25]

- **Sudden onset:** Symptoms typically begin within hours of drug exposure, with patients often able to recall the exact time and circumstances of onset.
- **Acute systemic symptoms:** Chills, fever, abdominal pain, diarrhoea, and nausea or vomiting have been described during exposure to the causative drug.
- **Anuria:** Rapid onset, typically within hours.
- **Neurologic findings:** These may range from mild confusion to coma.

Diagnostic evaluation

The diagnosis of OXL-related DITMA is primarily clinical, based on the presence of MAHA, thrombocytopenia, and organ dysfunction—normally kidney injury—following exposure to OXL. Laboratory findings include[23,24]:

- Thrombocytopenia, anaemia with elevated LDH, and low haptoglobin from scavenging of free haem.
- Visualisation of schistocytes in the peripheral blood smear.
- A compensatory increase in reticulocytes (to replace the lost red blood cells).
- Normal coagulation.
- A typically negative DAT.
- Renal insufficiency is common (characterised by elevated serum creatinine, proteinuria, and bland urine sediment).

Patients suspected of having OXL-related DITMA may benefit from testing for DDAbs, especially in cases where multiple drugs are involved and identifying the causative drug is necessary.

Tests to identify alternative causes of MAHA and thrombocytopenia should also be considered (See Table 2). ADAMTS13 activity is typically >20%, and complement dysregulation is usually absent, although nonspecific findings such as low C3 or C4 may be observed. Stool studies for Shiga toxin-producing *Escherichia coli* or other diarrhoeal pathogens are negative in DITMA.

Kidney biopsy is generally unnecessary for diagnosing DITMA or excluding other causes of TMA, but it may be considered if the cause of acute kidney injury remains unclear. When performed, a biopsy typically reveals TMA findings, such as platelet-rich thrombi in small arterioles and capillaries.

**TABLE 2. Useful tests in the investigation of MAHA and causes of thrombocytopenia**
Full blood count
Peripheral blood smear
Kidney function
Lactate dehydrogenase
Direct Antiglobulin Test
Coagulation testing with prothrombin time (PT) and activated partial thromboplastin time (aPTT)
ADAMTS13 activity level
Urinalysis
Stool testing for Shiga toxin (if diarrhoea is present)
Complement function

Differential diagnosis[15,24]

Clinicians must maintain a high index of vigilance for alternative causes of primary TMAs, microangiopathic haemolytic anaemia (MAHA), and thrombocytopenia. This is especially important given the significant clinical overlap with DITMA and the often subtle distinguishing features. Accurate differentiation is critical to ensuring appropriate management.

- **Primary TMA diagnoses:** Several primary TMA syndromes, such as thrombotic thrombocytopenic purpura (TTP), complement-mediated TMA, Shiga toxin-mediated haemolytic uremic syndrome (ST-HUS), and TMAs due to genetic defects in haemostasis or cobalamin metabolism, can mimic DITMA. These conditions frequently present as acute, life-threatening illnesses characterised by microangiopathic haemolysis, thrombocytopenia, and kidney damage. Specific diagnostic markers often differentiate these conditions from DITMA:
    - **Thrombotic Thrombocytopenic Purpura (TTP):** Severe ADAMTS13 deficiency (activity ≤10%) is a hallmark finding. Prompt recognition is essential as TTP requires urgent therapeutic plasma exchange (TPE) along with supportive therapy.
    - **Shiga toxin-mediated HUS (ST-HUS):** Identification of Shiga toxin-producing diarrheal organisms supports this diagnosis.

While these markers are crucial, their absence does not exclude these conditions, necessitating a thorough clinical evaluation.

- **Other forms of drug-induced thrombocytopenia:** Drug-induced thrombocytopenia can also present with temporal relationships to drug exposure but lacks the microangiopathic haemolysis and organ injury typical of DITMA. Examples include:
  - **Drug-Induced Immune Thrombocytopenia (DIITP):** Characterised by isolated thrombocytopenia without systemic involvement.
  - **Drug-Induced Thrombotic Thrombocytopenic Purpura (DI-TTP):** Though rare, this condition may mimic primary TTP but requires evidence of a temporal association with drug use.
  - **Drug-Induced Bone Marrow Suppression:** This typically manifests without features of MAHA or organ damage.
- **Other types of drug-induced anaemia:** Other anaemias linked to drug exposure include:
  - **Immune-Mediated Haemolysis:** Often presents with a positive DAT, in contrast to DITMA.
  - **Glucose-6-Phosphate Dehydrogenase (G6PD) Deficiency:** While this may be associated with haemolysis after drug exposure, it lacks fragmentation haemolysis, thrombocytopenia, or organ injury seen in DITMA.
- **Other types of drug-induced kidney injury:** Many drugs are potentially nephrotoxic, and it is crucial to differentiate direct kidney injury from the vascular injury associated with TMA. Like DITMA, drug-induced kidney injury may show a temporal association with drug exposure, rising serum creatinine, and sometimes anuria. Unlike DITMA, however, drug-induced nephrotoxicity generally does not include microangiopathic haemolysis or thrombocytopenia.

SECTION B. Re-exposure to OXL

Mandatory Criteria for Re-exposure to OXL:

1. Absolute oncological indication to continue treatment
2. No available therapeutic alternatives with comparable efficacy
3. Index reaction did not involve life-threatening compromise
4. Index reaction was followed by rapid recovery
5. Benefits of drug reexposure outweigh the risks of discontinuing treatment
6. Written informed consent obtained from the patient for re-exposure, following a thorough explanation of the risks, benefits, and alternative treatment options, and after addressing any questions or concerns

OXL Administration

OXL was administered as a 2-hour infusion in 5% dextrose solution. In patients with a confirmed history of type I hypersensitivity and positive skin testing results, a single-bag desensitisation protocol was used (see Section C).

Clinical Monitoring

The re-exposure to OXL was carried out in a dedicated, allergy-specific area within the oncology infusion unit, namely the Drug Hypersensitivity and Desensitisation Centre (DHDC). This centre operates under the direct supervision of the allergy department, ensuring that all procedures meet the highest standards of safety and care.

The allergy department meticulously coordinated the re-exposure process, overseeing the planning, risk assessment, and supervision of the protocol. Vital signs—including blood pressure, heart rate, respiratory rate, peripheral oxygen saturation, and body temperature—were continuously monitored at regular intervals using appropriate equipment. Automated alerts for abnormalities enabled prompt intervention.

A team of allergists specialising in drug allergy and desensitisation supervised the procedure, ensuring expert care throughout. One-to-one nursing care was provided by staff with expertise in allergy and drug desensitisation, delivering personalised and focused attention.

The procedure was conducted in a risk-assessed, high-dependency setting with immediate access to emergency support, ensuring prompt intervention if required. The allergist led the multidisciplinary team (MDT), which included specialists from medical oncology, intensive care, and oncology nursing. Each team member had a clearly defined role in managing potential complications, ensuring proactive

communication throughout the re-exposure process. The MDT worked in close collaboration with medical oncology, intensive care, and other relevant services to provide continuous updates on the patient's condition and management plan.

The patient was kept fully informed throughout the procedure, with clear communication about the risks and each step involved. A dedicated nurse provided emotional support, ensuring the patient remained comfortable and reassured. Regular structured briefings and updates kept all staff informed, promoting a coordinated and well-integrated approach to care.

Upon completion of the infusion, the patient was transferred to the ward for a minimum 24-hour observation period, during which vital signs, blood tests, and urine output were closely monitored for any signs of adverse reactions. Discharge was considered only if no adverse events occurred during this time. Before discharge, the patient was provided with a comprehensive Patient Information Leaflet (PIL), outlining potential symptoms of delayed reactions, guidance on follow-up care, emergency contact details, and the importance of promptly reporting any concerns.

In the event of any adverse reactions during the re-exposure process, immediate intervention was carried out in line with established protocols. The MDT responded swiftly, ensuring that any reactions were treated promptly and effectively. All interventions were thoroughly documented to ensure compliance with institutional protocols and regulatory standards for drug desensitisation procedures. Following the procedure, a structured debriefing was conducted, during which the MDT reviewed the patient's response, discussed any challenges faced, and identified opportunities to refine protocols to improve future care. This debriefing process is essential for continuous quality improvement, ensuring that each procedure is safer and more efficient.

Serial Laboratory Monitoring

Serial laboratory tests were conducted at baseline and at 2, 8, 20, 46 hours, and 5-7 days following the OXL infusion. In the event of a reaction, hypersensitivity biomarkers were incorporated into the laboratory analysis.

See detailed list in in Table 3:

TABLE 3. Routinely monitoring upon a suspected II-HSR	
**Test**	Timing
Full blood count<br>Reticulocytes<br>Peripheral blood smear<br>Creatinine<br>Glomerular filtration rate<br>Direct bilirubin<br>Total bilirubin<br>Alanine transaminase<br>Aspartate transaminase<br>Ferritin<br>Haptoglobin<br>Lactate dehydrogenase (LDH)<br>C Reactive Protein (CRP)<br>Procalcitonin (PCT)<br>D-Dimer<br>Fibrinogen<br>Activated partial thromboplastin time (aPTT)<br>Partial thromboplastin time (PTP)<br>Direct antiglobulin test (DAT)<br>Urine Sediment	Baseline and 2h, 8h, 20h, 46h and 5-7 days post OXL-infusion
**In case of BTR**	
**Biomarker**	**Timing**
Tryptase	1h and 2h after-BTR
Interleucin-6	0 min, 60 min, 2h, 8h, 20h and 46h after-BTR

SECTION C. One Bag RDD protocol

Example of the standard-flexible ICO-HUB one bag RDD protocol for a total dose of 200mg of OXL meant to be infused in a volume of 500 ml over 2 hours.

Drug: oxaliplatin — **Total volume in the bag:** 500ml

Total dose: 200 mg — **Drug concentration in the bag:** 0.4 mg/ml

Step	Rate (ml/h)	Time per step (min)	Administered volume (ml)	Administered dose (mg)	Fold increase per step (mg/min)	Cumulative dose infused (mg)
**1**	1	15	0.25	0.1	NA	0.1
**2**	2	15	0.5	0.2	x2	0.3
**3**	4	15	1	0.4	x2	0.7
**4**	8	15	2	0.8	x2	1.5
**5**	16	15	4	1.6	x2	3.1
**6**	32	15	8	3.2	x2	6.3
**7**	48	15	12	4.8	x1.5	11.1
**8**	72	15	18	7.2	x1.5	18.3
**9**	106	15	27	10.8	x1.5	29.1
**10**	162	15	40.5	16.2	x1.5	45.3
**11**	250	93	386.75	154.7	x1.5	200

Total infusion time: 243 min (4 hours and 3 minutes)

Safety considerations: In line with international guidelines, this RDD protocol should only be utilised by expert allergists in allergy-dedicated spaces that are properly equipped and staffed to manage these high-risk, high-complexity procedures.

Premedication: This is a premedication-sparing, one-bag protocol. However, premedication should follow the manufacturer's instructions and institutional protocols for standard infusion of each specific drug. We do not recommend additional systemic premedication (e.g., steroids or antihistamines) to prevent breakthrough reactions, especially for the first procedure. Tailored premedication may be considered on a case-by-case basis, particularly for patients who have experienced breakthrough reactions during previous RDD procedures at our centre.

Adjustments to the volume of the bags: The standard volume for solution bags in the ICO-HUB protocol is 500 ml, provided this is consistent with the manufacturer's instructions. In certain cases, the bag volume may need to be adjusted based on the manufacturer's guidance or product information.

Simultaneous diluting fluids: A 500 mL saline 0.9% bag (or glucose 5% for platinum-based drugs) is infused simultaneously through the same line (dual lumen). The flow rate for the fluids is programmed according to the same protocol as the drug, preventing potential errors that could arise from having different flow rates for the drug and fluids. This ensures that the dilution of the culprit drug remains consistent throughout the process.

Adjustments to the fold increase between steps: The fold increase is x2 up to step 6, after which it changes to x1.5. Different fold increases between steps may be used when personalising protocols, such as in reactive patients where a more cautious increase could be applied to reactive steps, or when a minor adjustment is made to simplify protocol calculations. Fold increases between steps should always remain within the recommendations outlined by rapid drug desensitisation guidelines.

Adjustments to final infusion rate: Step 11 may be adjusted to achieve the desired final infusion rate. The final infusion rate can be tailored to align with the standard regimens specified by the referring oncologist or personalised for reactive patients (e.g., by using a slower final infusion rate). Additionally, extra steps may be incorporated to reach higher infusion rates, ensuring that the dose increase between each step remains within a range of 1.25-fold to 2.5-fold.

Infusion pumps: Precision infusion pumps should be used to ensure accurate adherence to the protocol, which involves very small volumes at most steps. These pumps should be equipped with automatic multi-step infusion capabilities to minimise human errors that may arise from manually adjusting infusion rates every 15 minutes.

Legend: NA, not applicable; RDD, rapid drug desensitisation; ICO, Catalan Institute of Oncology, Barcelona, Spain; HUB, Bellvitge University Hospital, Barcelona, Spain.

Reprinted from Delabeling patients from chemotherapy and biologics allergy: Implementing drug provocation testing, J Allergy Clin Immunol Pract. Apr;9(4):1742-1745., Copyright (2021), with permission from Elsevier.

SECTION C. Oncological Tumour features

TABLE 4. Oncological tumour features from patients diagnosed with II-HSRs												
**Case**	**Sex**	**Date of cancer Dx**	**Age at cancer Dx**	**Stage at Dx**	**Primary tumor**	**Molecular profile**	**Adj. OXL**	**Adj. OXL cycles**	**Other previous regimens**	**Setting at II-HSR**	**Cum. OXL cycles**	**Cum. OXL dose (mg)**
**1**	F	24/7/2015	57	IIIB	Sigmoid	RAS wt / BRAF wt / MSS	Yes	10	Perioperative 5-FU	First line	19	NA
**2**	F	2/11/2015	51	IVA	Rectum	KRAS G12D / MSS	Yes	8	FOLFIRI	First line maintenance	32	4,472
**3**	F	2/7/2019	59	IVB	Rectum	RAS wt / HER2 amplified / MSS	No	NA	FOLFIRI + Cetuximab	Rechallenge	15	2,162
**4**	M	11/6/2019	47	IVB	Sigmoid	RAS wt / BRAF wt / MSS	No	NA	None	First line maintenance	20	3,367
**5**	M	6/9/2022	64	IVB	Sigmoid	KRAS G12A / MSS	No	NA	None	First line maintenance	20	2,338
**6**	M	7/2/2019	50	IVB	Sigmoid	KRAS G12D / MSS	No	NA	FOLFIRI + Bevacizumab	Rechallenge	13	2,078
**7**	M	6/2/2017	61	IIIB	Rectum	KRAS G13D / MSS	Yes	8	FOLFIRI + Aflibercept	Rechallenge	22	3,842
**8**	M	29/3/2017	60	IIIC	Sigmoid	RAS wt / BRAF wt / MSS	Yes	10	FOLFIRI + Cetuximab	Rechallenge	13	2,076
**9**	F	17/2/2016	58	IIIB	Sigmoid	RAS wt / BRAF wt / MSS	Yes	11	FOLFIRI + Cetuximab	Rechallenge	18	2,557
**10**	M	2/12/2016	68	IVB	Sigmoid	RAS wt / BRAF wt / MSS	No	NA	FOLFIRI	Rechallenge	15	1,617
**11**	M	7/6/2018	67	IVB	Ascending	KRAS G12A / MSS	No	NA	FOLFIRI	First line maintenance	21	NA
**12**	M	15/11/2016	55	IIIB	Ascending	KRAS G13D / MSS	Yes	12	FOLFIRI + Bevacizumab, TAS102	Rechallenge	20	NA
**13**	M	4/10/2013	58	IIIB	Rectum	KRAS A146T / MSS	Yes	8	FOLFIRI	Rechallenge	19	2,383
**14**	M	2/9/2014	61	IIIA	Sigmoid	RAS wt / BRAF wt / MSS	Yes	12	None	Rechallenge	19	NA
**15**	M	1/7/2020	57	IIIB	Jejunum	KRAS Q61H /MSS	Yes	8	FOLFIRI + Aflibercept	Rechallenge	35	4,947
**16**	M	10/5/2021	60	IVA	Rectum	KRAS G12V / MSS	No	NA	None	Rechallenge	19	2,333

F: female, M: male, Dx: diagnosis, NA: not available / not applicable, wt: wild type, 5-FU: 5-fluorouracil, FOLFOX: leucovorin, fluorouracil and oxaliplatin, FOLFIRI: leucovorin, fluorouracil and irinotecan

References

1. Danese E, Montagnana M, Favaloro EJ, Lippi G. Drug-Induced Thrombocytopenia: Mechanisms and Laboratory Diagnostics. *Semin Thromb Hemost*. 2020;46(3):264-274. doi:10.1055/S-0039-1697930
2. Stack A, Khanal R, Denlinger CS. Oxaliplatin-induced Immune Thrombocytopenia: A Case Report and Literature Review. *Clin Colorectal Cancer*. 2021;20(1):e1-e4. doi:10.1016/J.CLCC.2020.07.007
3. Jardim DL, Rodrigues CA, Novis YAS, Rocha VG, Hoff PM. Oxaliplatin-related thrombocytopenia. *Annals of Oncology*. 2012;23(8):1937-1942. doi:10.1093/ANNONC/MDS074
4. Bencardino K, Mauri G, Amatu A, et al. Oxaliplatin Immune-Induced Syndrome Occurs With Cumulative Administration and Rechallenge: Single Institution Series and Systematic Review Study. *Clin Colorectal Cancer*. 15(3):213-234. doi:10.1016/j.clcc.2016.02.001
5. Cobo F, De Celis G, Pereira A, Latorre X, Pujadas J, Albiol S. Oxaliplatin-induced immune hemolytic anemia: a case report and review of the literature. *Anticancer Drugs*. 2007;18(8):973-976. doi:10.1097/CAD.0B013E3280E9496D
6. MA B, WT S, CS C, BR C, RH A, CT H. Hypersensitivity reaction and acute immune-mediated thrombocytopenia from oxaliplatin: two case reports and a review of the literature. *J Hematol Oncol*. 2010;3. doi:10.1186/1756-8722-3-12
7. Heikal NM, Smock KJ. Laboratory testing for platelet antibodies. *Am J Hematol*. 2013;88(9):818-821. doi:10.1002/AJH.23503
8. Danese E, Montagnana M, Favaloro EJ, Lippi G. Drug-Induced Thrombocytopenia: Mechanisms and Laboratory Diagnostics. *Semin Thromb Hemost*. 2020;46(3):264-274. doi:10.1055/S-0039-1697930
9. Garbe E, Andersohn F, Bronder E, et al. Drug induced immune haemolytic anaemia in the Berlin Case-Control Surveillance Study. *Br J Haematol*. 2011;154(5):644-653. doi:10.1111/J.1365-2141.2011.08784.X
10. Arndt PA, Garratty G. The changing spectrum of drug-induced immune hemolytic anemia. *Semin Hematol*. 2005;42(3):137-144. doi:10.1053/j.seminhematol.2005.04.004
11. Barcellini W, Fattizzo B. Clinical Applications of Hemolytic Markers in the Differential Diagnosis and Management of Hemolytic Anemia. *Dis Markers*. 2015;2015. doi:10.1155/2015/635670
12. Iams W, Beckermann KE, Neff AT, Mayer IA, Abramson VG. Thrombotic microangiopathy during docetaxel, trastuzumab, and carboplatin chemotherapy for early-stage HER2+ breast cancer: A case report. *Medical Oncology*. 2013;30(2). doi:10.1007/S12032-013-0568-X
13. PA M, M B, C B, et al. Making the Correct Diagnosis in Thrombotic Microangiopathy: A Narrative Review. *Can J Kidney Health Dis*. 2021;8. doi:10.1177/20543581211008707
14. Siau K, Varughese M. Thrombotic microangiopathy following docetaxel and trastuzumab chemotherapy: a case report. *Med Oncol*. 2010;27(4):1057-1059. doi:10.1007/S12032-009-9333-6
15. Saleem R, Reese JA, George JN. Drug-induced thrombotic microangiopathy: An updated systematic review, 2014-2018. *Am J Hematol*. 2018;93(9):E241-E243. doi:10.1002/AJH.25208
16. Lucchesi A, Carloni S, Cangini D, Frassineti GL, Casadei Gardini A. Acute oxaliplatin-induced thrombotic thrombocytopenic purpura: A case report and results from a cytoflourimetric assay of platelet fibrinogen receptor. *Journal of Clinical Oncology*. 2013;31(16):2061-2062. doi:10.1200/JCO.2012.48.3248/ASSET/IMAGES/ZLJ9991034110001.JPEG
17. Phan NT, Heng AE, Lautrette A, Kémény JL, Souweine B. Oxaliplatin-induced acute renal failure presenting clinically as thrombotic microangiopathy: think of acute tubular necrosis. *NDT Plus*. 2009;2(3):254-256. doi:10.1093/NDTPLUS/SFP008
18. Niu J, Mims MP. Oxaliplatin-induced thrombotic thrombocytopenic purpura: case report and literature review. *J Clin Oncol*. 2012;30(31). doi:10.1200/JCO.2012.42.5082
19. Saad R, Hannun A, Temraz S, Finianos A, Zeenny RM. Oxaliplatin-induced thrombotic microangiopathy: a case report. *J Med Case Rep*. 2022;16(1). doi:10.1186/S13256-022-03309-7
20. Arndt PA, Leger RM, Garratty G. Serology of antibodies to second- and third-generation cephalosporins associated with immune hemolytic anemia and/or positive direct antiglobulin tests. *Transfusion (Paris)*. 1999;39(11-12):1239-1246. doi:10.1046/J.1537-2995.1999.39111239.X
21. Norton A, Roberts I. Management of Evans syndrome. *Br J Haematol*. 2006;132(2):125-137. doi:10.1111/J.1365-2141.2005.05809.X
22. Fattizzo B, Michel M, Giannotta JA, et al. Evans syndrome in adults: an observational multicenter study. *Blood Adv*. 2021;5(24):5468-5478. doi:10.1182/BLOODADVANCES.2021005610
23. Hanna RM, Henriksen K, Kalantar-Zadeh K, Ferrey A, Burwick R, Jhaveri KD. Thrombotic Microangiopathy Syndromes-Common Ground and Distinct Frontiers. *Adv Chronic Kidney Dis*. 2022;29(2):149-160.e1. doi:10.1053/J.ACKD.2021.11.006
24. Brocklebank V, Wood KM, Kavanagh D. Thrombotic Microangiopathy and the Kidney. *Clin J Am Soc Nephrol*. 2017;13(2):300. doi:10.2215/CJN.00620117
25. JN G, JM M, NW L, CM N. After the Party's Over. *N Engl J Med*. 2017;376(1):54. doi:10.1056/NEJMCPS1606750
